# Mesoscale structures in signed networks

**DOI:** 10.1126/sciadv.aee5886

**Published:** 2026-10-09

**Authors:** Wei Zhang, Olga Boichak, Tristram J. Alexander, Tiago P. Peixoto, Eduardo G. Altmann

**Affiliations:** ^1^ School of Mathematics and Statistics, University of Sydney, Sydney, 2006 NSW, Australia.; ^2^ Centre for Complex Systems, University of Sydney, Sydney, 2006 NSW, Australia.; ^3^ School of Art, Communication, and English, University of Sydney, Sydney, 2006 NSW, Australia.; ^4^ School of Physics, University of Sydney, Sydney, 2006 NSW, Australia.; ^5^ Center for Critical Computational Studies, Goethe University Frankfurt, 60629 Frankfurt am Main, Germany.; ^6^ Institute of Computer Science, Goethe University Frankfurt, 60629 Frankfurt am Main, Germany.; ^7^ IT:U Interdisciplinary Transformation University, 4040 Linz, Austria.; ^8^ Department of Network and Data Science, Central European University, 1100 Vienna, Austria.; ^9^ Complexity Science Hub, 1030 Vienna, Austria.

## Abstract

Mesoscale structures in signed networks have been studied under the limiting assumption of the validity of social balance theory, which predicts positive connections within groups and negative connections between groups. Here, we propose and apply a methodology that overcomes this limitation and is able to find and characterize also the different possible unbalanced structures in signed networks. Applying our methodology to 24 empirical networks, from social-political, financial, and biological domains, we find that unbalanced mesoscale structures are prevalent in real-world networks, including cases with substantial balance at the microscale of triangles. In particular, we find that assortativity often prevails regardless of the interaction sign and that core-periphery structures are typical in online social networks. Our findings highlight the complexity of mesoscale relational structures, the importance of using computational methods that are a priori agnostic to specific patterns, and the importance of independently evaluating micro- and mesoscale predictions of social balance theory.

## INTRODUCTION

The recent renewed interest in signed networks (*1*–*9*) reflects the need for network representations of complex phenomena to distinguish between opposing types of interactions. For instance, online interactions among users (vertices) do not always reflect friendship or agreement (positive edges) and are thus better described through networks that account for enmity or disagreement (negative edges). Representing these relationships as signed networks allows researchers not only to capture the presence and strength of connections among vertices but also differentiate the effects of positive and negative interactions on the structure of the network. Large signed networks typically show a rich combination not only of microscale (e.g., triadic interactions) but also of mesoscale structures (e.g., communities), which are known to be essential to understanding the underlying complex system (e.g., the partition of users in opposing groups) (*10*–*13*).

Signed networks have been primarily studied within structural balance theory (*14*), which originated in studies of social and economic networks (*15*). A signed network is considered balanced (antibalanced) if and only if all cycles have an even number of negative (positive) edges (*14*, *16*, *17*). In the simplest case of three cycles, the signed network with all triads having zero or two negative edges is balanced. While this microscale perspective of structural balance theory is the most popular, structural balance can also be equivalently viewed from a mesoscale perspective: Balanced (antibalanced) signed networks can be partitioned into two communities whose intralinks are all positive (negative) and interlinks are all negative (positive) (*16*, *18*). In a weaker version, k-balance theory states that networks are balanced if they can be partitioned into k≥2 communities with all intracommunity links being positive and all intercommunity links being negative (*7*, *8*, *19*). These balance conditions are rarely satisfied strictly in signed networks obtained from empirical datasets. Approaches to quantify the extent into which a signed network is balanced involve the comparison to suitable null models (*5*, *20*) of balance measures computed at different network scales (*4*, *21*). At a microscale, the degree of balance (DoB) is computed on the basis of local configurations, including triadic or motif analysis (*14*, *22*); at a mesoscale, the focus shifts to the aggregate structure of the network and its distance from a perfectly balanced state, quantified by the frustration index (FI) (*6*, *20*, *23*); structural balance can also be viewed from a dynamical perspective and quantified using the spectrum of the signed Laplacian (*24*, *25*) and walk-based measure (*26*, *27*).

A critical step in the mesoscale approach mentioned above is the partitioning of the network into groups. Different community detection methods, originally proposed for simple networks, have been recently extended to signed networks: modularity maximization (*11*, *12*, *28*–*31*), spectral clustering (*24*, *25*, *32*, *33*), and spin glass (*34*, *35*). The primary goal of these methods is to find a partition that is close to a k-balanced partition, i.e., to divide the network into separate communities so that nodes within the same community have as many positive connections as possible, while nodes in different communities have as many negative connections as possible. These descriptive partitioning methods are designed to optimize for specific structural objectives (*4*, *11*, *21*) and can succeed when structural balance is the dominant feature shaping the mesoscale organization of the network. However, a substantial challenge arises when the goal is the unbiased detection of mesoscale structures without the a priori assumption of structural balance. Because these methods are constrained by their objective functions, they may impose balanced partitions even when the data are better described by alternative patterns or, as we show below, even in random networks where balanced structures are absent. It is thus unclear in which extent the reported balanced structures are the dominant mesoscale feature of the network or simply a consequence of the assumptions made by the methods. Since mesoscale structures in signed networks have been studied almost exclusively within structural balance theory, several fundamental questions remain open: To what extent are the mesoscale structures of real-world signed networks aligned with structural balance theory? Are there different types of unbalanced structures, and, if so, what do they reveal about the organization of large-scale signed networks?

In this work, we propose and apply a framework to overcome the limitations of previous approaches and to answer these questions. Our goal is to find, characterize, and quantify the strength of mesoscale structures in signed networks beyond the balance assumption. We achieve this by proposing an exhaustive pairwise classification of structures in signed graphs—generalizing previous classifications for unsigned graphs (*36*, *37*) and connecting this classification to structural balance theory—and combining it with state-of-the-art inferential methods for community detection (*38*), which have already highlighted that diverse mixing patterns may simultaneously coexist in a network (*6*, *39*–*43*). In particular, we use stochastic block models (SBMs) (*13*, *38*, *44*–*46*) that leverage statistical evidence and do not a priori adhere to specific types of balanced community structures. Applying this framework to 24 empirical datasets, we find that unbalanced structures are present in almost all networks and are dominant in a significant portion of them. In particular, we find that social networks are often best partitioned into groups in which positive and negative links happen within the same group, possibly reflecting the interaction opportunities available to individuals. We also find that relationships akin to core-periphery structures are widespread in (the negative edges of) online social networks. Last, we show how the micro- and mesoscale characterization of structural-balance theory often leads to complementary pictures of the processes shaping complex signed networks.

## RESULTS

### Methodology

We first consider how mesoscale partitions of signed networks are related to structural balance theory at its usual microscale representation. Figure 1 shows a simple case connecting the two scales, with balanced and antibalanced mesoscale communities fulfilling the microscale definitions based on triads. A balanced network shows diagonal blocks in the positive edge density matrix and off-diagonal blocks in the negative edge density matrix (Fig. 1B, bottom). This indicates that balanced communities exhibit assortative relationships in positive interactions and disassortative relationships in negative interactions (*36*, *37*). Conversely, antibalanced communities show disassortative relationships in positive interactions and assortative relationships in negative interactions. This motivates us to use the edge density matrix to measure and classify diverse community structures, including unbalanced ones.

**Fig. 1.**
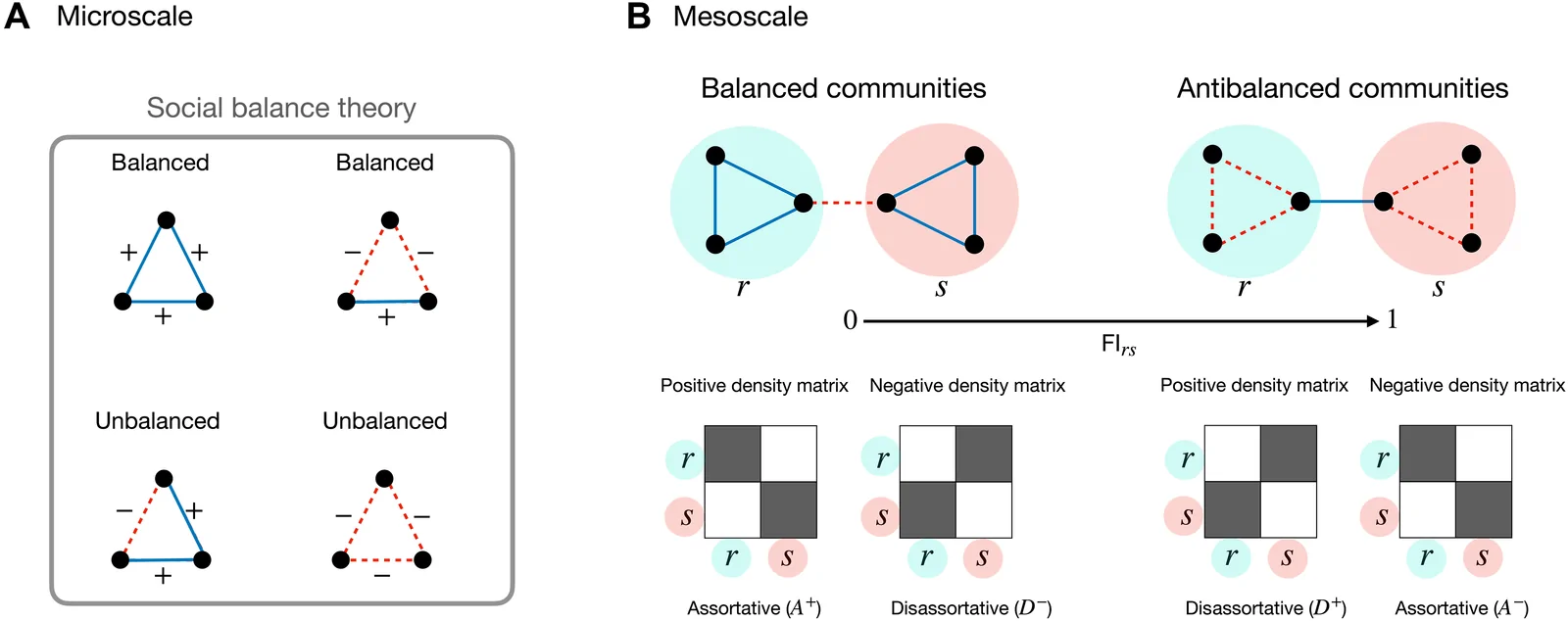
Structural balance theory from microscale and mesoscale perspectives. (**A**) Fundamental triads or motifs considered as balanced and unbalanced by social balance theory. (**B**) Balanced (left) and antibalanced (right) communities. Top: Simple signed networks, with positive links as solid lines and negative links as dashed lines. The two groups *r* and *s* are marked with circles. Bottom: The corresponding edge density matrices (*1*).

Consider a simple undirected signed graph *G* with adjacency matrix $\left(A_{\mathrm{ij}}\right)\in {\mathbb{R}}^{N\times N}$, where each entry $A_{\mathrm{ij}}$ can be written as the sum of two disjoint components $A_{\mathrm{ij}}=A_{\mathrm{ij}}^{+}+A_{\mathrm{ij}}^{-}$, with $A_{\mathrm{ij}}^{+}>0$ representing positive edges and $A_{\mathrm{ij}}^{-}<0$ representing negative edges. The graph is partitioned into r=1,2,3,…,B disjoint subgroups, each containing $N_{r}$ nodes. For a pair of communities *r* and *s*, the density of positive interactions and negative interactions is given by the ratio of existing edges and possible edges as

$$w_{\mathrm{rs}}^{\pm }=\left\{ \begin{array}{cc} \sum_{i\in r,j\in s}\frac{|A_{\mathrm{ij}}^{\pm }|}{N_{r}N_{s}}, & \text{if}\;r\neq s,\\ \sum_{i\in r,j\in s}\frac{|A_{\mathrm{ij}}^{\pm }|}{N_{r}\left(N_{s}-1\right)}, & \text{if}\;r=s \end{array}\right.$$ (1)

We now classify the relationship between a pair of communities *r* and *s* based on $w_{\mathrm{rs}}^{\pm }$. In simple graphs, as shown in Fig. 2A, this relationship can be classified as either assortative, core periphery, or disassortative (*36*, *37*, *47*). In signed networks, these relationships apply to both positive and negative edges

$$\left\{ \begin{array}{cc} \text{Assortative}\;\left(A^{\pm }\right), & \text{if}\;w_{\mathrm{rs}}^{\pm }<\text{min}\left(w_{\mathrm{rr}}^{\pm },w_{\mathrm{ss}}^{\pm }\right),\\ \text{Core}-\text{periphery}\;\left(C^{\pm }\right), & \text{if}\;\text{min}\left(w_{\mathrm{rr}}^{\pm },w_{\mathrm{ss}}^{\pm }\right)<w_{\mathrm{rs}}^{\pm }<\text{max}\left(w_{\mathrm{rr}}^{\pm },w_{\mathrm{ss}}^{\pm }\right),\\ \text{Disassortative}\;\left(D^{\pm }\right), & \text{if}\;w_{\mathrm{rs}}^{\pm }>\text{max}\left(w_{\mathrm{rr}}^{\pm },w_{\mathrm{ss}}^{\pm }\right) \end{array}\right.$$ (2)

**Fig. 2.**
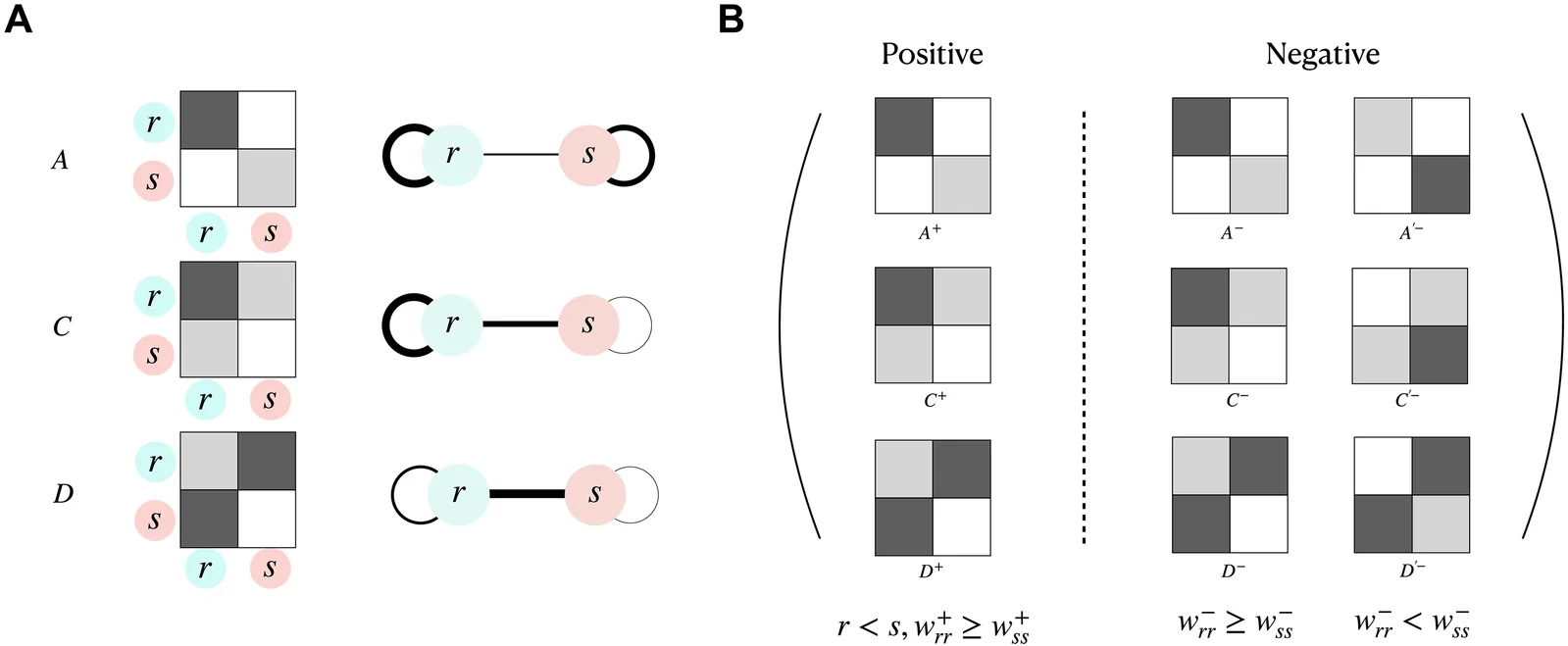
Pairwise classification of community types. (**A**) The three potential pairwise-community relations between communities *r* and *s* are represented by density matrices. The darker blocks indicate a higher density of edges. Assortative $A^{\pm }$ (disassortative $D^{\pm }$) cases have the two largest entries in the diagonal $w_{\mathrm{rr}}^{\pm }$, $w_{\mathrm{ss}}^{\pm }$ (off-diagonal $w_{\mathrm{rs}}^{\pm }$,$w_{\mathrm{sr}}^{\pm }$). The core-periphery type of relationship $C^{\pm }$ has one sparser community (periphery) linked strongly to the denser community (core), which is in turn more interconnected within itself. (**B**) The three possible types for positive links and six for negative links, thus leading to a total of 18 possible combination types. Note that blocks are arranged such that the left column has the highest density of positive intrablock edges. This freedom is unavailable for the negative edges, so there are twice as many types.

In the classification above, we adopt the community label convention that $w_{\mathrm{rr}}^{\pm }\geq w_{\mathrm{ss}}^{\pm }$ for r<s. By applying this convention to $w^{+}$, this reduces the number of ranked relationships for the positive edges to three (shown in Fig. 2A), while six options remain for negative matrix $w^{-}$ (shown in Fig. 2B), because the rankings of $w_{\mathrm{rr}}^{-}$ and $w_{\mathrm{ss}}^{-}$ may be oppositely ordered. To differentiate the two options (i.e., $w_{\mathrm{rr}}^{-}>w_{\mathrm{ss}}^{-}$ and $w_{\mathrm{rr}}^{-}<w_{\mathrm{ss}}^{-}$), we label this latter configuration by a prime symbol ′. For instance, the pair $\left(C^{+},C^{\prime -}\right)$ signifies that the two communities are in a core-periphery relationship in both positive and negative edges but that the community that acts as core is different in each of the edge types (the notation $\left(C^{+},C^{-}\right)$ represents the relationship in which both cores are the same community). When no distinction between these cases is intended, we denote the relationship as $\left(C^{+},C^{\left(\prime \right)-}\right)$. To quantify mesoscale balance, we define a structural scale connecting the two theoretical extremes: Balanced ($A^{+},D^{\left(\prime \right)-}$) and antibalanced ($D^{+},A^{\left(\prime \right)-}$). Each pairwise relationship type is assigned a score: +1 for assortative (*A*), −1 for disassortative (*D*), and 0 for core-periphery (*C*) in the case of positive interactions (these values are reversed for negative interactions). Thus, there are a total of 3×6=18 possible relationships between two communities *r* and *s* for signed networks. These relationships can be grouped into balanced, unbalanced, and antibalanced groups, as shown in Fig. 3.

**Fig. 3.**
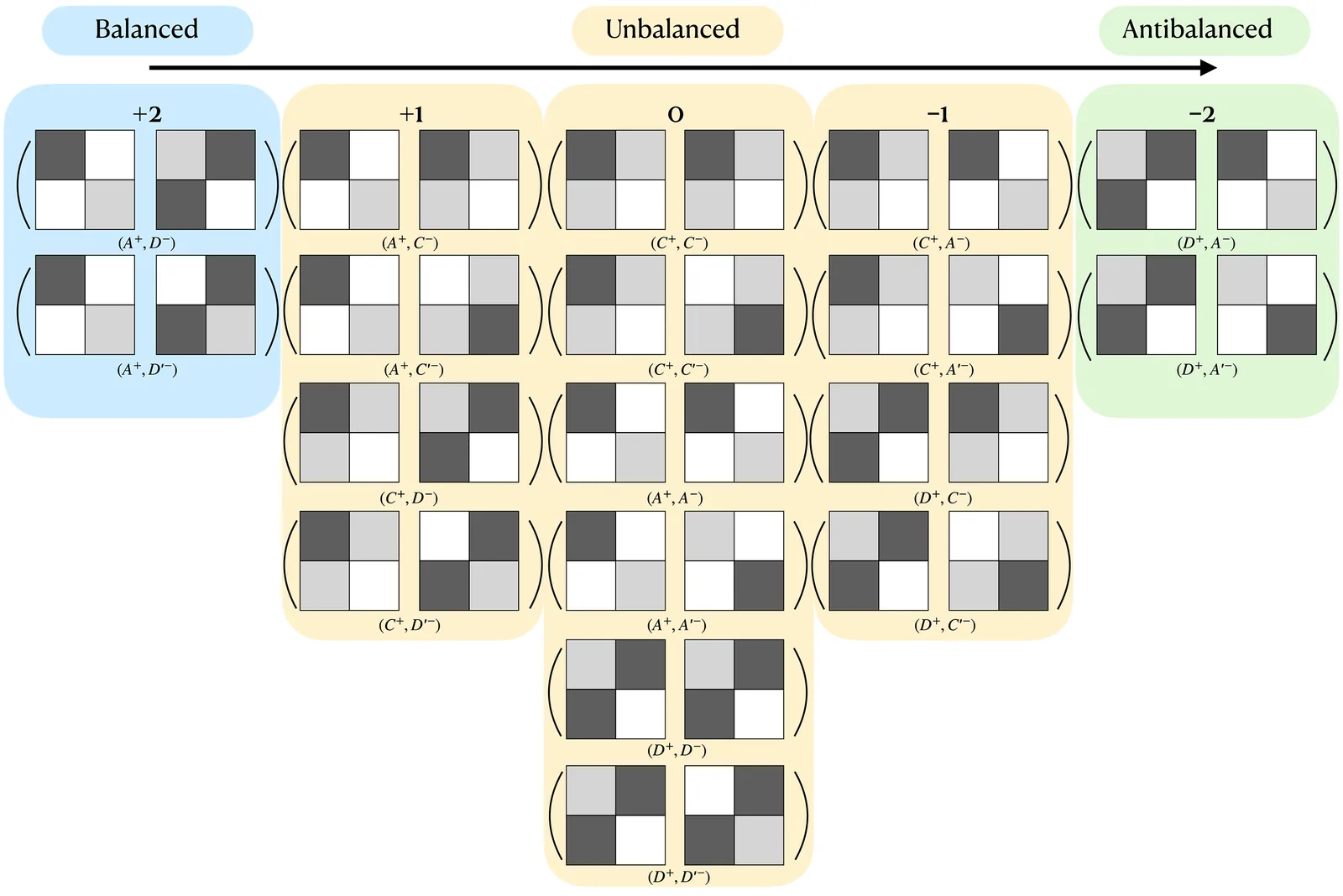
Pairwise classification of mesoscale structures in signed networks. $A^{\pm }$, $C^{\pm }$, and $D^{\pm }$ stand for “assortative,” “core-periphery,” and “disassortative” in positive (+) or negative (−) interaction, respectively, and are defined by Eq. 2. For example, the pair $\left(A^{+},D^{\prime -}\right)$ represents that the positive edges are assortative and the negative edges are disassortative, and the mark ′ labeled in $D^{\prime -}$ indicates that the negative diagonal entries $w_{\mathrm{rr}}^{-}<w_{\mathrm{ss}}^{-}$. From left to right, the 18 different possible configurations are divided according to their level of balance. A relationship is balanced when positive edges are assortative (dense within communities) and negative edges are disassortative (dense between communities). In contrast, a structure is antibalanced when positive edges are disassortative and negative edges are assortative. These balanced (antibalanced) structures are considered to have weak structural balance (antibalance) since they allow the limited presence of other entries in the density matrix. The remaining unbalanced configurations are scored as follows: assortative is +1, core-periphery is 0, and disassortative is −1. For negative edges, the scoring is as follows: disassortative is +1, core-periphery is 0, and assortative is −1.

Our classification serves as building blocks to analyze the structural profile of a signed network. To apply the pairwise classification described above to a network with B≥2 communities, we need to (i) identify the groups of nodes (i.e., partition the network into *B* communities) and (ii) classify each of the $\left(\begin{array}{c} B\\ 2 \end{array}\right)$ pairs’ relation type into one of the 18 possible combinations, as shown in Fig. 4. Different methods for network partition can produce varying B×B density matrices, and our methodology can be applied to any of these partition methods.

**Fig. 4.**
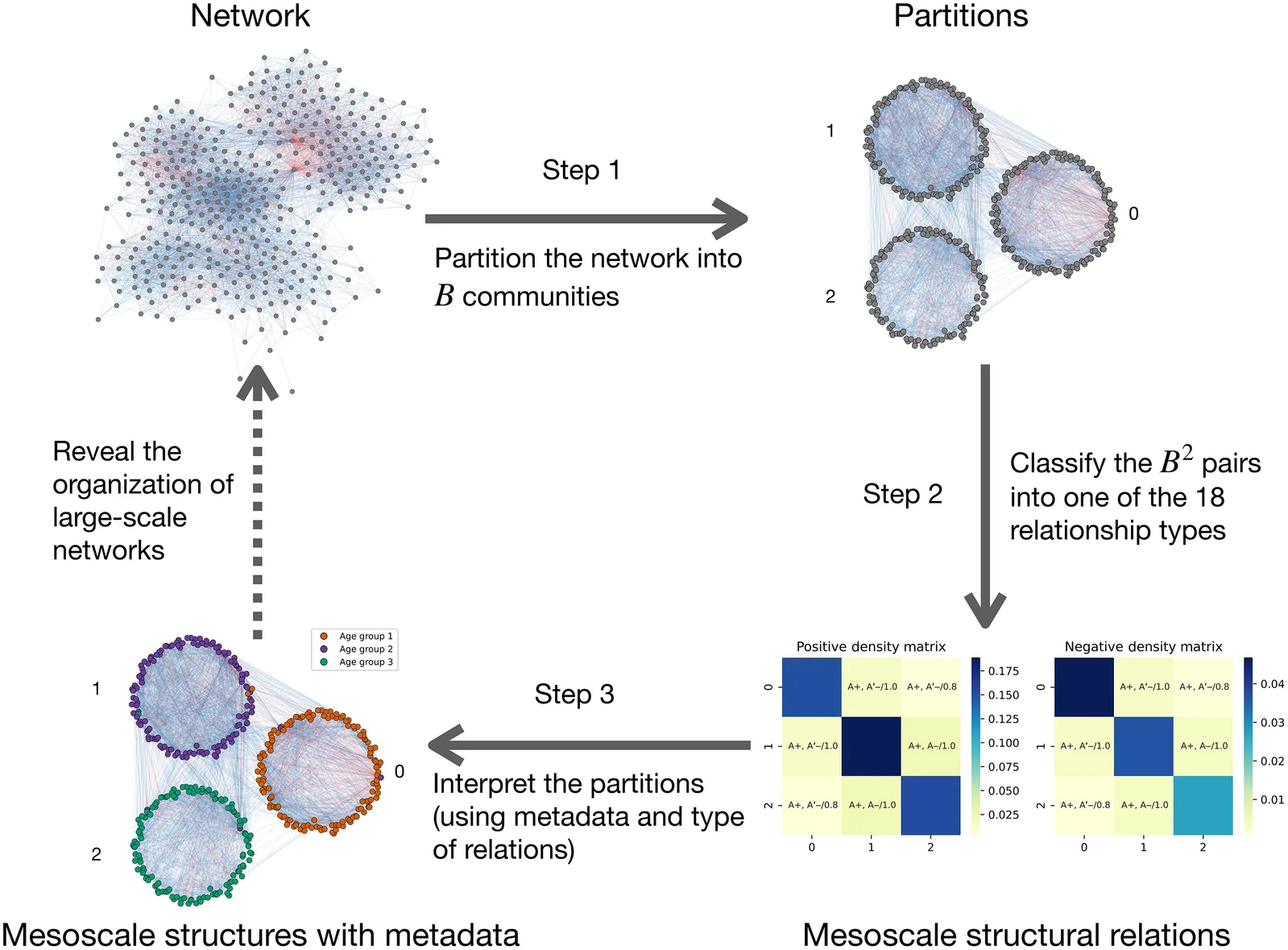
Illustration of the main steps of our methodology. Step 1: The signed network is partitioned into *B* communities using a suitable partition method, such as the ones listed in Table 1. Step 2: Each pair of communities is classified according to its type, as proposed in Fig. 3. Step 3: This classification is then used—in combination with metadata, when available—to interpret the mesoscale structures and quantify the DoB at a mesoscale level.

The results presented here primarily involve five methods, including traditional methods that focus on balanced partitions—Louvain (*30*, *31*), spectral clustering (*25*), and spin glass (*11*)—and inferential approaches that are agnostic about it—layered SBM (LSBM) (*13*) and weighted SBM (WSBM) (*45*) (for comparison of the methods, see Table 1). Hierarchical versions of SBMs (*48*) can also be directly used in combination with our classification by analyzing pairwise relationships at different hierarchy levels. We then analyze each of the $\left(\begin{array}{c} B\\ 2 \end{array}\right)$ pairs, we classify their relationship type into one of the 18 possible combinations, and we focus on whether a specific relationship appears in that network (contributing to the overall prevalence of the type) and which type is the most common (dominant).

**Table 1.** List of methods used for the partition of signed networks. The table compares five methods according to their core idea, whether they assume structural balance, their computational complexity, and their empirical runtime on three example networks of various sizes: (i) *N* = 446 with 13,908 positive and 36,780 negative edges; (ii) N=3883 with 2808 positive and 2672 negative edges; (iii) N=8711 with 22,910 positive and 2163 negative edges) using a laptop (with an Apple M3 chip, 16 GB of memory, running macOS 26.3 25D125). For smaller numbers of groups B, methods with higher asymptotic complexity (e.g., spectral clustering) can run faster than inferential alternatives; see (*38*).

Property	LSBM (*13*)	WSBM (*45*)	Louvain (*30*, *31*)	Spectral (*25*)	Spin glass (*11*)
Core idea	Inference: Two-layer shared partition.	Inference: Block-pair distributions.	Modularity: Iterative modularity maximization.	Linear algebra: Maximizes internal (+), external (−) edges.	Statistical-physics: Spin alignment via edge signs.
Balance assumption?	No	No	Yes	Yes	Yes
Complexity	$O\left(N{\text{log}}^{2}N\right)$	$O\left(N{\text{log}}^{2}N\right)$	O(NlogN)	$O\left(N^{2}\text{log}N\right)$	–
Running time in seconds (number of groups *B*)					
Network i	3.64 s (19)	2.78 s (27)	0.10 s (3)	0.23 s (2)	40.25 s (12)
Network ii	2.88 s (4)	2.26 s (8)	0.06 s (1911)	3.47 s (2)	27.00 s (50)
Network iii	8.39 s (35)	8.96 s (36)	0.17 s (325)	24.36 s (13)	27.00 s (49)

### Unbalanced structures are common

We now apply the methodology described in “Methodology” above to empirical networks. We collected 24 empirical signed networks, which include 18 social-political networks, 2 financial networks, and 4 gene-regulatory networks (see Materials and Methods section “Data preprocessing” and the Supplementary Materials for a detailed description of the dataset). These networks vary substantially in size, between 16 and 10,884 nodes and between 40 and 251,406 edges (see table S2 for a list).

The results obtained across all networks are summarized in Fig. 5. As expected, the dominant relationship type in most networks for the balanced-based methods (e.g., Louvain and spin glass) is the balanced structure $\left(A^{+},D^{\left(\prime \right)-}\right)$, with few unbalanced structures detected except for $\left(A^{+},A^{\left(\prime \right)-}\right)$. This reflects the fact that these methods aim to find balanced partitions, thus providing little information about the type of structure present in the data. The results obtained for the two inferential methods (i.e., LSBM and WSBM), which do not focus exclusively on balanced partitions and rely instead on statistical evidence, find more unbalanced structures with the dominant type of relationship over all networks being the unbalanced structure $\left(A^{+},A^{\left(\prime \right)-}\right)$ and with different core-periphery relationships observed as well. Similar results are observed when we restrict the analysis to social-political networks, financial networks, and gene-regulatory networks (figs. S1 to S3). Notably, nonsocial mechanisms shape structure differently from social networks and are known to show lower degrees of balance (*23*). For gene-regulatory networks, our method shows (fig. S2) that both SBM methods identify unbalanced structural relationships to be dominant in all cases, contrary to the Louvain method that detects balanced structures as dominant in all cases. This finding indicates that unbalanced structures among communities are not just a theoretical possibility; they are actually common in empirical networks provided that suitable partition methods are used.

**Fig. 5.**
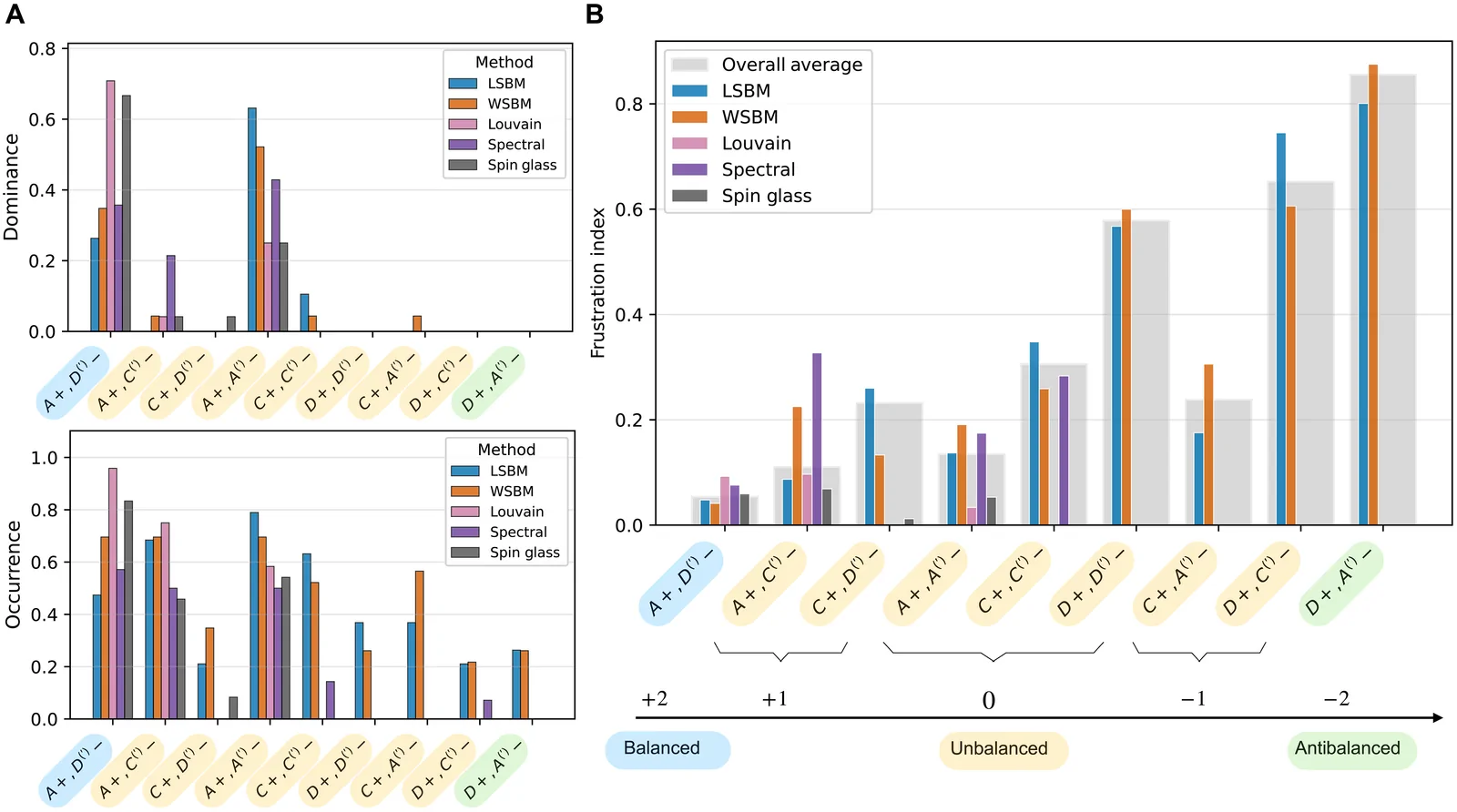
Survey on 24 empirical networks. (**A**) Community types in empirical networks. Top: Fraction of networks in which each community type is dominant (computed as the proportion of networks in which the type is the most prevalent). Bottom: Fraction of networks in which each community type occurs (computed as the proportion of networks in which the type appears at least once). (**B**) FI in empirical networks. The *x* axis represents balance categories ranging from balanced, unbalanced, to antibalanced configurations, while the *y* axis shows the FI. Bars correspond to different methods, with the overall average shown in gray. The calculation of the pairwise FI refers to Materials and Methods section “Frustration index”.

We validate our pairwise approach by comparing the consistency of the relationships we found with traditional methods from structural balance theory. We compare our classification of mesoscale structure types to the average pairwise FI, which measures the fraction of misplaced links, positive interlinks, and negative intralinks, between communities (see Materials and Methods section “Frustration index” for details). An FI=0 indicates perfect balance, while FI=1 indicates complete antibalance. The results in Fig. 5B reveal a clear trend: Balanced structures correspond to low frustration, unbalanced structures exhibit intermediate levels, and antibalanced structures show the highest frustration. This progression is consistent across different methods, and the overall averages align closely with theoretical expectations, confirming the alignment of our classification scheme to structural balance theory.

Below, we explore the significance and interpretation of these unbalanced relationships, focusing on the partitions obtained using SBMs. This choice is motivated by their ability to detect such patterns as the underlying generative network model can, in principle, contain any of the pairwise relationship types discussed previously. We use degree-corrected SBMs as a generative process to prevent the classification of high-degree nodes into blocks based solely on their connectivity (*44*). The number of blocks (communities) *B* and the block allocation of each node are obtained by performing Bayesian inference, following the approach proposed in (*46*, *49*, *50*). Sampling the posterior distribution over partitions allows us to evaluate the robustness of the proposed partitions and of the classification of the mesoscale structures, including the extent into which they are balanced (see Materials and Methods section “SBM inference” for details). The results of posterior samplings (tables S3 and S4 and fig. S5) confirm that unbalanced structures were predominant. We compared the dominant types identified in our survey with those from posterior sampling. For networks with dominant-type certainty >0.5, the consistency rate was 100% for LSBM and 88% for WSBM; across all networks, it was 96 and 79%, respectively. Inconsistencies arise from two main factors: low posterior certainty, indicating alternative community structures, and cases where the survey-identified type is the second most frequent, with a frequency close to that of the dominant type (fig. S6).

### Assortativity prevails regardless of interaction sign

In most empirical networks investigated in the section “Unbalanced structures are common,” the dominant type found using SBMs is the unbalanced structure $\left(A^{+},A^{\left(\prime \right)-}\right)$, while for other methods, the dominant type is the balanced structure $\left(A^{+},D^{\left(\prime \right)-}\right)$. To understand this difference and the origin of $\left(A^{+},A^{\left(\prime \right)-}\right)$, we now focus on the example of the relationship between students across the three years of high school in a Spanish school. The results in Fig. 6 show that, without using any age information, the partitions obtained from SBMs align with the three distinct age groups with resulting assortative relationships between any two groups, in both positive and negative interactions $\left(A^{+},A^{\left(\prime \right)-}\right)$. The robustness of this interaction type and our conclusions is further confirmed by our posterior sampling analysis (see table S5). This illustrates a pattern where links are driven by the interaction of students within their age groups, regardless of the signs of the ties, while interactions between different age groups are relatively scarce. While social homophily is recognized as the foundational mechanism of connectivity in positive-only networks (*51*), our findings reveal that negative interactions adhere to the same structural logic. In comparison, the groups detected by Louvain involve a mixture of age groups and are mostly in balanced relationships ($A^{+},D^{\left(\prime \right)-}$) with each other.

**Fig. 6.**
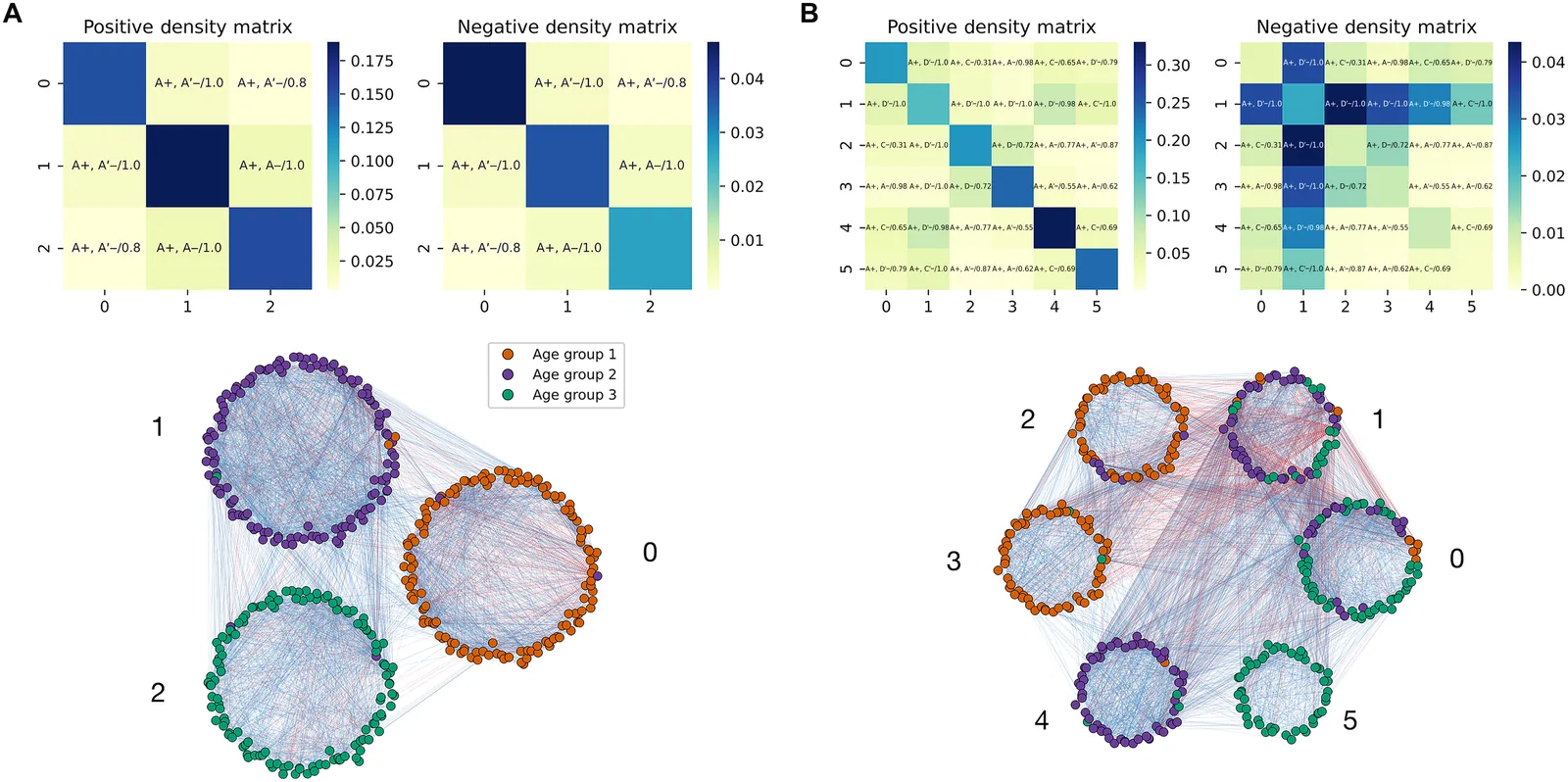
Partition of Spanish High School network with WSBM and Louvain. Network partitions obtained using (**A**) WSBM and (**B**) the Louvain method. Top: The blocks are ranked according to community size. The labels in each entry of the matrix correspond to the structure type and the robustness score (see Materials and Methods section “Classification robustness”). Bottom: The communities are grouped closely together, and the numbers denote the labels of each group. The colors of the nodes represent different age group memberships.

To validate this finding, we generate synthetic random networks mimicking high-school interactions. We consider three equal-sized age groups with positive edges predominantly within the group and negative edges controlled by a tunable parameter. Our results in Fig. 7 show that, when the negative edges are across different groups, all methods correctly identify the (ground truth) balanced partition. Increasing the number of negative edges within groups, the SBM methods succeed in retrieving the three groups, but the heuristic methods have a much more limited success. Most strikingly, the Louvain method finds a larger number of groups that show balanced relationships, in line with the observations in the Spanish high-school data. Together, these results show that assortativity (regardless of interaction sign) is a prevailing pattern in signed networks. This pattern is masked by the use of heuristic descriptive methods (such as Louvain), which find balanced network partitions $\left(A^{+},D^{\left(\prime \right)-}\right)$ that can be wrongly interpreted as a confirmation of the predictions of social balance theory.

**Fig. 7.**
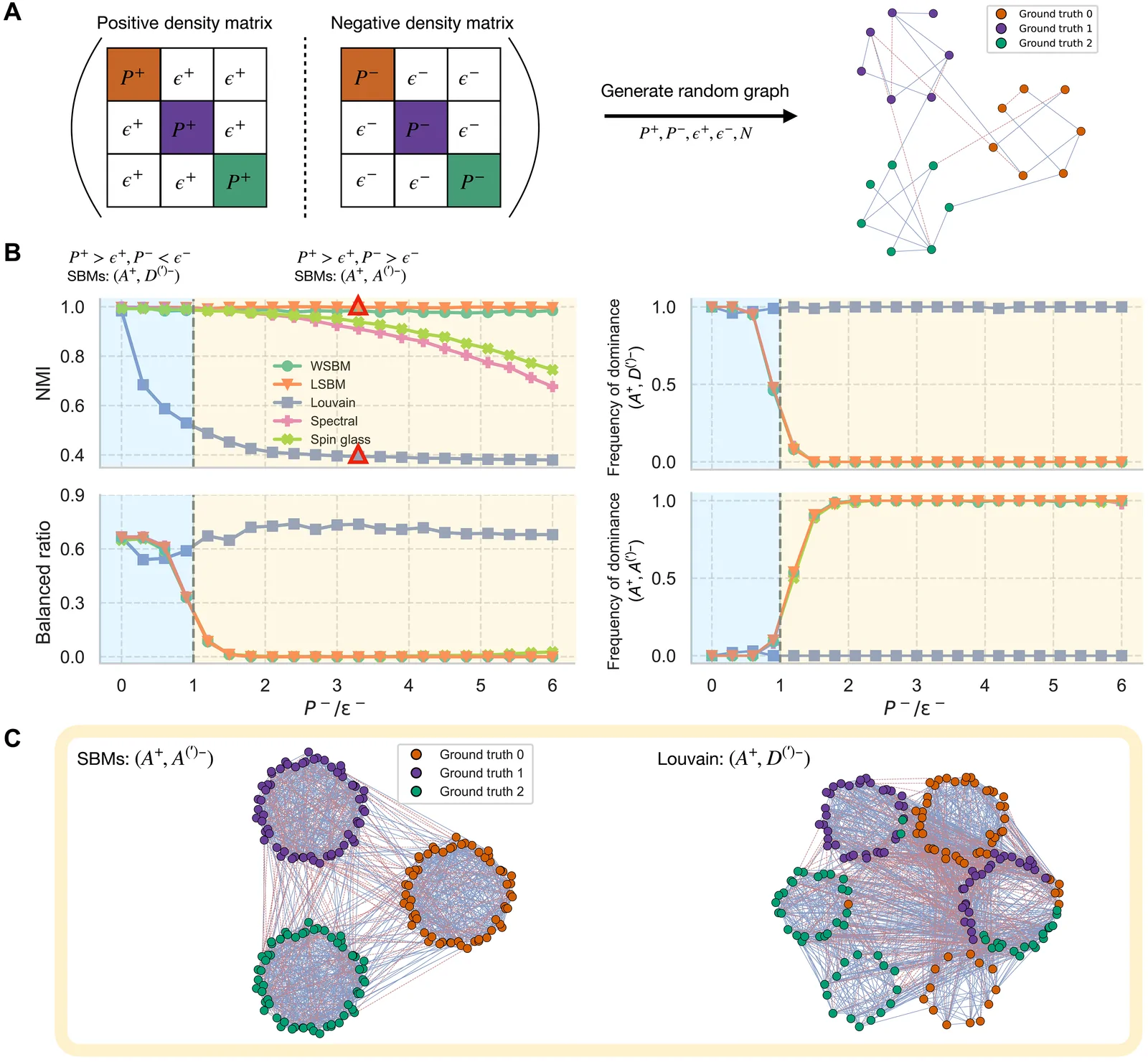
Heuristic methods lead to biased partitions in unbalanced synthetic networks. (**A**) Generative model schematic with three ground-truth groups with equal size (N/3 nodes each). The intragroup interaction probability is $P^{+}$ (positive) and $P^{-}$ (negative), and the intergroup probabilities are $\epsilon^{+}$ (positive) and $\epsilon^{-}$ (negative). Nodes are color-coded by group, with solid blue lines and red dashed lines representing positive and negative links, respectively. For $P^{-}<\epsilon^{-}$, the network is dominated by $\left(A^{+},D^{\left(\prime \right)-}\right)$ relationships (balanced regime) while for $P^{-}>\epsilon^{-}$ by $\left(A^{+},A^{\left(\prime \right)-}\right)$ relationships. (**B**) Performance of different network-partition methods against the ground truth. NMI (Materials and Methods) and balanced ratio $\left(A^{+},D^{\left(\prime \right)-}\right)$ (left), and fraction of random networks identifying specific dominant interaction types (right), across 100 independent synthetic random networks. The dashed gray line ($P^{-}=\epsilon^{-}$) marks the theoretical boundary between the balanced (blue) and unbalanced (yellow) regimes. SBMs correctly identify the transition from balanced to unbalanced dominance, matching the generative ground truth. In contrast, Louvain always finds balanced structures, even when the ground truth is unbalanced. (**C**) Example of partitions obtained by SBM and Louvain, with nodes colored according to the ground truth. The parameters used in all plots are $P^{+}=0.2,\epsilon^{+}=\epsilon^{-}=0.01,N=180$. In (C), $P^{-}=0.033$ was used [red triangle in (B)].

A possible origin of $\left(A^{+},A^{\left(\prime \right)-}\right)$ structures, such as the one reported in the Spanish High School and synthetic network, is a network with assortative communities with edge signs added independently of the mesoscale structure. In this trivial case, the mesoscale partition would be retrieved using classical community-detection methods (e.g., classical Louvain). To test whether such a trivial explanation applies in our case, we used description length to compare our WSBM model of sign graphs with a simpler SBM model for the unsigned graph (see Materials and Methods section “Model selection” for details). The results obtained in the Spanish High School indicate that the signed-graph WSBM is overwhelmingly preferred, indicating that despite the prevailing $\left(A^{+},A^{\left(\prime \right)-}\right)$ structure, there is still significant alignment between node connectivity and edge signs at the mesoscale level. Applying this comparison to the 24 networks used in our survey, we find that 18 of them (75%) show this preference, while in 6 (25%), the simpler model of unsigned SBM is preferred (see fig. S7). This shows that edge signs are highly informative about the mesoscale organization in most empirical networks, including cases with both balanced and unbalanced partitions.

### Core periphery is typical in social networks

Another frequent unbalanced structure in our survey of empirical networks in section “Unbalanced structures are common” is the core-periphery structure. Traditionally, in such structures, the core represents a dense cluster of nodes with strong internal connectivity, while the periphery consists of loosely connected nodes that rely on the core for interaction (*37*, *47*). For unsigned interactions in social media, the core typically comprises influencers or highly active users who act as information creators, whereas the periphery primarily consumes information (*47*, *52*, *53*). While core-periphery structures have been extensively studied for positive or neutral interactions, their role in negative exchanges remains less clear. Our results reveal a distinct and previously underreported behavior of the core under negative interactions, which we detail in Fig. 8 and below in three empirical examples:

**Fig. 8.**
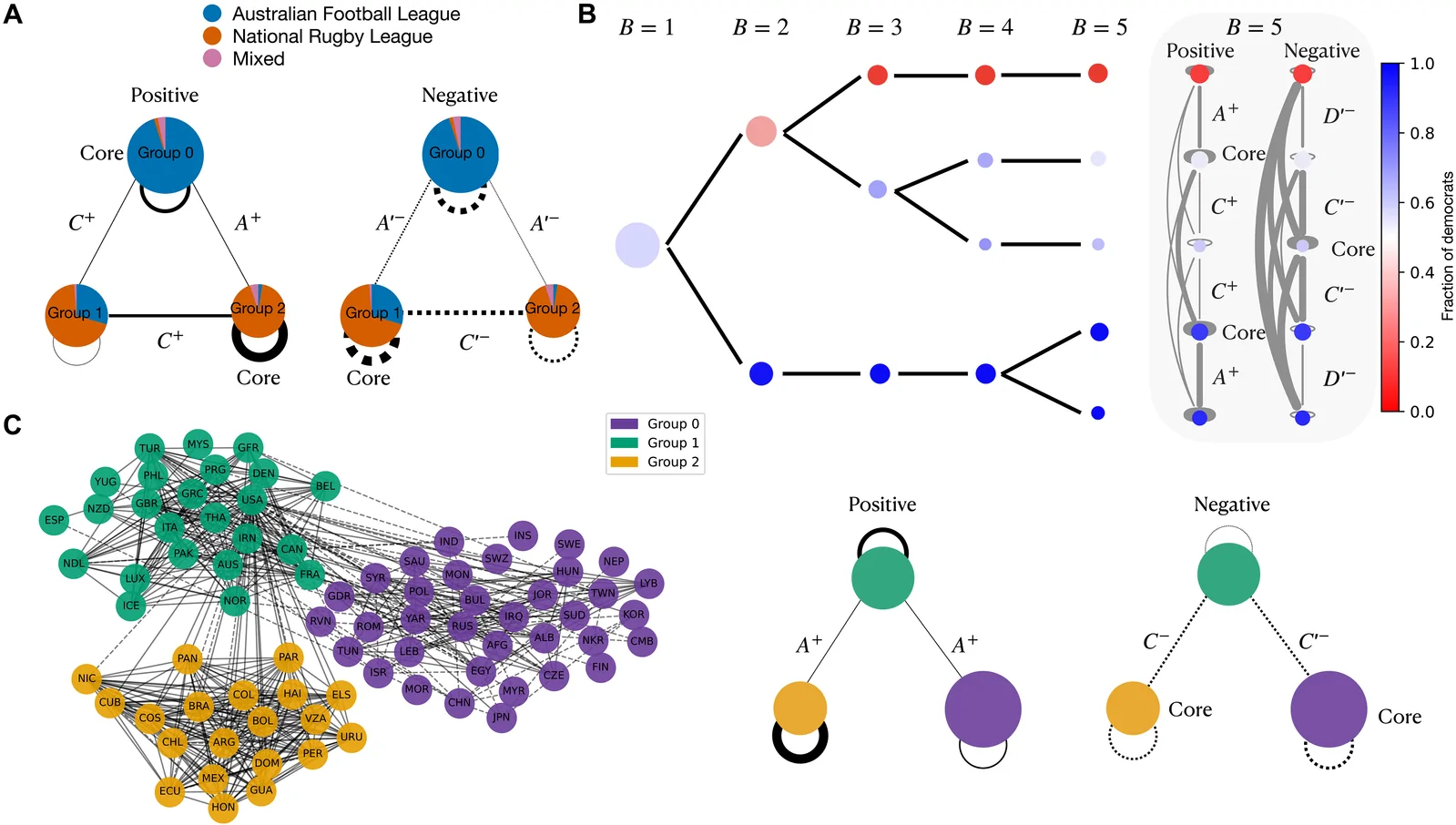
Examples of core-periphery structures in signed networks. (**A**) Twitter signed network (*3*). The graphical representation of the edge density matrix corresponding to the three top-level groups identified by WSBM. Each circle (node) represents a community, and the colored sectors within each node illustrate the topic composition: Australian Football League (AFL), National Rugby League (NRL), and mixed-interest discussions. (**B**) USA Congress H100 (*54*). The partition into groups was obtained by the LSBM method with an increasing number of communities B∈[0,5]. The plot shows schematically how communities divide and the type of pairwise interaction they share. The diagram on the right displays the positive and negative density matrix for *B* = 5. Colors indicate party affiliation of group members. (**C**) Correlates of Wars for the years 1954 to 1957 (*55*). It shows the top-level network partitioned by WSBM (left) and the graphical representation of the edge density matrix for the communities (median and right). The label on each node is the name of the region. In all plots, the size of each node reflects the size of the group, solid lines represent positive edges, and dashed lines represent negative edges.

1) A social-media network of 3883 Twitter users discussing sports in Australia, with positive edges representing “like” and negative edges representing opposing replies (*3*). Our analysis of the network partition (Fig. 8A) reveals two core-periphery structures in positive interactions and one core-periphery structure in negative interactions. By mapping the topical interests of users (metadata) to the inferred blocks, we identify that groups 0 and 2 are each dominated by users mostly interested in different sports (Australian Football League and National Rugby League, respectively). As expected in these cases, the relationship between these two groups in the positive network is assortative, characterized by high internal positive edge densities. Conversely, group 1 emerges as a mixed-topic hub. While it occupies a peripheral role in positive interactions, lacking a singular sports identity to drive cohesive likes, it is a central core role in the negative interactions. This suggests that groups containing members of diverse interests or opinions bridge otherwise assortative topical clusters (*53*), serving as a critical structural bridge that sustains discourse across the entire network. We note that a posterior analysis of this network revealed plausible alternative partitions of the network, including types of mesoscale structures different from the one reported here (see table S6).

2) A well-known political network of the Congress of the United States of America (USA), constructed on the basis of the cosponsorship patterns of bills in the 100th House of Representatives (*54*). Nodes are members of the House of Representatives, and edges represent significant positive or negative tendencies to cosponsor bills. Although our network partition (Fig. 8B) was obtained without using party affiliation metadata, the inferred blocks align closely with partisan lines, providing a structural explanation for legislative behavior. While the balanced structure is dominant, we focus on the emergence of core-periphery structures that reveal more interesting patterns (confirmed by our posterior sampling analysis, see table S7). For positive interactions, the cores are composed of highly cohesive partisan blocks (e.g., relatively solid Democrat or Republican clusters) that exert influence over peripheral groups containing more mixed members of both parties. Most significantly, the negative interactions reveal a mixed-party core. While balance theory might predict that negative ties only exist between party blocks, we find that bipartisan clusters, containing both Democrats and Republicans, serve as the structural core of the negative interactions.

3) The well-studied Correlates of War network for the years 1954 to 1957 (*55*). Nodes represent geopolitical regions, and edges denote formal alliances (+) or diplomatic/military conflicts (−). The partition (Fig. 8C) reveals three main groups in assortative relationships in the positive interactions but in core-periphery relationships in the negative interactions (the robustness of this pattern is confirmed by our posterior sampling analysis, see table S8). While group 1 (comprising the USA and Western Europe) remains internally cohesive through a dense positive alliance, its broader global connectivity is primarily mediated through negative interactions. Specifically, its engagement with the rest of the world is defined by prominent negative edges with the conflictual cores. In this structure, group 0, a geographically diverse cluster spanning Europe, Africa, and Asia, emerges as the primary negative core. This indicates that group 0 functions as the central arena of global friction, connected with the established western bloc (group 1).

These examples reveal a common pattern: Core-periphery relationships often appear in the negative interactions with negative cores often composed by diverse groups that accommodate internal conflicts (becoming the main arena for conflict), sit at the center of disputes, and connect different groups. In other words, they act as mixed hubs that concentrate antagonistic ties and mediate interactions across opposing factions. These structures are not accidental but functional. Reference (*53*) points out that core-periphery formations are topologically optimized for the efficient diffusion of specialized information from an active core to a relatively passive periphery. In the context of negative interactions in social networks, our results suggest that this specialized information often takes the form of controversial or polarizing discourse coming from groups with members with different affiliations. Thus, these unbalanced structures act as structural bridges that concentrate disagreement at the center to maintain network-wide engagement, suggesting that they are critical for understanding the dynamics of negotiation and disagreement in social and political networks (*53*, *56*).

### Micro and mesoscales exhibit different balance

Structural balance is a scale-dependent property (*27*). Although perfectly balanced or antibalanced states are equivalent across micro and mesoscales under strong balance theory (Fig. 1), measuring imperfect balance reveals important differences. We quantify balance at two levels: local triadic balance (DoB) at the microscale and overall frustration at the mesoscale. Contrary to the expectation that local balance implies global balance, Fig. 9A shows only a weak correlation between them (i.e., a weak negative correlation between the microscale DoB and the mesoscale frustration). This result indicates that microscale and mesoscale balance are not interchangeable: Local structural balance does not reliably predict global frustration. To isolate the effect of local structure from this broader mesoscale organization, we introduce null models sampled from SBMs as a baseline for comparison (see Materials and Methods section “Null-model testing”). These null models preserve the mesoscale block structures and edge sign densities, allowing us to compute the *z* score for observed DoB. As shown in Fig. 9B, most empirical networks have a significantly higher microscale balance than expected under the fixed mesoscale structures. Overall, these results confirm that the micro-level and meso-level balance are distinct dimensions of signed network structure, with networks with similar triad balance exhibiting vastly different levels of mesoscale balance (overall frustration).

**Fig. 9.**
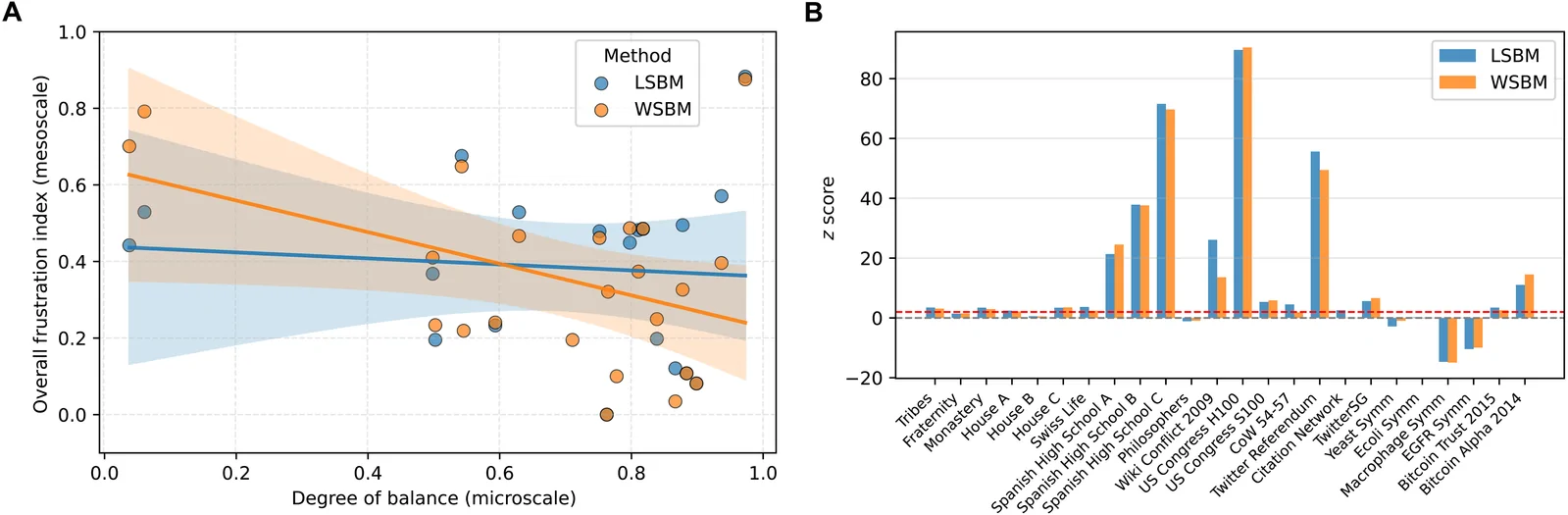
Microscale balance versus mesoscale frustration. (**A**) Negative relationship between DoB (fraction of balanced triads) and overall FI. Scatter plots show data points of each network with linear regression fits and 95% confidence intervals (shaded regions). (WSBM: $R^{2}=0.181,P=0.043$; LSBM: $R^{2}=0.008,P=0.708$). (**B**) *Z* scores quantify the deviation of observed DoB from null models preserving mesoscale block structure. The red dashed line is when *z* score = 2. Even when mesoscale structure is fixed, microscale balance is significantly overrepresented in real networks, indicating that microlevel balance and mesolevel balance are not interchangeable. Other relationships between mesoscale (im)balance and microscale balance also support this result (fig. S4). The computation of DoB and *z* score refers to Materials and Methods sections “Balanced triads” and “Null-model testing.”

## DISCUSSION

Social balance theory has been a cornerstone of social network analysis for over half a century (*14*, *15*), providing a foundational framework for the study of signed networks, where positive ties denote friendship or alliance and negative ties represent enmity or conflict. Traditionally, quantitative studies of signed networks have focused on micro-level triad analysis (e.g., “the friend of my friend is my friend” and “the enemy of my enemy is my friend”). While this micro-level analysis has provided valuable insights, it has an emphasis on local configurations and overlooks the complexity of the connectivity present in empirical networks, which includes different mesoscale structures. To address this gap, we proposed and applied a methodology to characterize mesoscale structures in signed networks that does not a priori assume that structures are balanced, being thus suitable to evaluate the extent into which the observations are explained by structural balance theory and to reveal previously unidentified (unbalanced) structures shaping the network.

Applying this methodology to 24 empirical networks, including social, political, citation, financial, and biological networks, we find that unbalanced mesoscale structures are ubiquitous, even when substantial balance exists at the microscale of triangles. In many empirical networks, particularly social and political ones, the dominant structure is not balanced but instead assortative in both positive and negative interactions. The case study of Spanish High School students illustrates such a pattern of social homophily in both positive and negative interactions (*51*): The frequency of interaction often outweighs the sign of the connection; individuals interact within their social clusters regardless of the nature of the tie. In contrast, as confirmed by our study on synthetic networks (Fig. 7), methods that are constrained to detect balanced partitions fail to capture this (and other) unbalanced relationships even when they are the prevailing mesoscale structure of signed networks. While the particular synthetic case discussed above focused on $\left(A^{+},A^{\left(\prime \right)-}\right)$ structures, which can potentially be recovered applying classical community-detection methods (e.g., Louvain), ignoring edge signs, this is not the case for other unbalanced structures (see fig. S8 for a similar analysis focusing on synthetic graphs with unbalanced nonassortative structures). In particular, only 4 of the 18 pairwise structures we define here (in Fig. 3) are detected using traditional heuristic methods: the two balanced ones—$\left(A^{+},D^{-}\right)$ and $\left(A^{+},D^{\prime -}\right)$—using methods based on structural balance (e.g., signed Louvain), and the two assortative ones—$\left(A^{+},A^{-}\right)$ and $\left(A^{+},A^{\prime -}\right)$—using community-detection methods (e.g., traditional Louvain). The remaining 14 structure types are invisible for these methods, requiring the combination of the inferential and classification approaches proposed here to be detected and interpreted. The lack of detection of nonbalanced and nonassortative structures by heuristic methods is not due to detectability limits, which are present also in signed graphs [as studied in (*57*)]. Instead, it is due to the embedded assumptions of assortativity and balance in these methods, rendering them systematically blind to broader classes of mesoscale organizations.

Another important finding of our work is that core-periphery structures play an important role in signed social networks. This includes not only the expected cases of well-connected influencers who create content consumed by the periphery (*47*, *52*) but also a new pattern in which a conflict-tolerant or mixed-affiliation group constitutes the core of a negative core-periphery relationship. While distinct, this negative core-periphery structure is theoretically consistent with prior research suggesting that core-periphery formations are topologically optimized for the efficient diffusion of specialized information from an active core to a relatively passive periphery (*53*). In this context, we posit that polarized discourse and intergroup conflict serve as the specialized information, with the mixed-group core acting as the primary generator of high-engagement, controversial, or diverse content. Consequently, the distinction between core structures in positive versus negative interactions serves to demarcate divergent behavioral patterns among users.

Future research should question assumptions of balance and consider the presence of unbalanced mesoscale structures in network representations. As our findings suggest, we can reasonably expect that studies with signed networks in other areas will be similarly successful in identifying diverse structures and providing new insights not only on the role of specific nodes but also on the organization and function of mesoscale communities and the network as a whole. In particular, the application of our results to online social-media debates has the potential to reveal mesoscale structures beyond opinion polarization between two groups. These analyses may require a generalization of our methods to directed and weighted signed networks (*37*, *58*) (i.e., where influence may not be reciprocal) with the usage of more general SBMs and moving beyond a simple pairwise classification of community types to capture more multifactional structures.

## MATERIALS AND METHODS

### Data preprocessing

We consider 18 social-political networks, 4 gene-regulatory networks, and 2 financial networks. A basic description of these networks can be found in the Supplementary Materials. While some networks have directed (e.g., Spanish High School and TwitterSG) and weighted (e.g., Spanish High School and Monastery) edges, here, networks were standardized by mapping the existing data to undirected (symmetrized) and unweighted (binarized) signed graphs with a single connected component. This was done to ensure a uniform comparison across all methods—while the SBMs can handle weighted directed edges, Louvain and spin glass cannot—and to align with structural balance theory in its standard form, which prioritizes the tie sign consistency over strength [but see (*58*) for a possible generalization of balanced triads to directed networks]. This was achieved by first considering only nodes in the largest connected component and then following the rules proposed in (*23*, *59*): If two nodes share the same sign in both directions, the undirected connection preserves that sign; if the connection is nonreciprocal, we symmetrize by adopting the existing sign; if the signs are opposite, the undirected edge is negative. For graphs with a weighted configuration, we binarized the weights by assigning +1 to each positive edge and −1 to each negative edge.

Formally, given a directed signed adjacency matrix $S=\left(S_{\mathrm{ij}}\right)\in {\mathbb{R}}^{N\times N}$ where $S_{\mathrm{ij}}>0$ indicating a positive edge, $S_{\mathrm{ij}}<0$ a negative edge, and $S_{\mathrm{ij}}=0$ no edge, we construct a simple undirected signed adjacency matrix $A=\left(A_{\mathrm{ij}}\right)\in {\mathbb{R}}^{N\times N}$, using the symmetrization rule

$$A_{\mathrm{ij}}=\left\{ \begin{array}{cc} 0, & S_{\mathrm{ij}}=0\;\text{and }S_{\mathrm{ji}}=0,\\ -1, & S_{\mathrm{ij}}S_{\mathrm{ji}}<0,\\ \text{sgn}\left(S_{\mathrm{ij}}+S_{\mathrm{ji}}\right), & \text{otherwise} \end{array}\right.$$ (3)

where sgn(x)=+1 if x>0, and sgn(x)=−1 if x<0.

Before preprocessing, we analyzed the prevalence of reciprocated edges and conflicting edges (i.e., directed edges between the same two nodes with opposing signs) among the directed networks we collected; see details for each network in table S1. Conflicting edges are extremely rare across all datasets: 50% of the directed networks have no conflicting edges, and the other 50% have less than 5% conflicting edges. After this preprocessing, we calculated the descriptive statistics for the largest connected component of each network. The results are summarized in table S2.

### Frustration index

To measure the structural (im)balance on the mesoscopic scale, we use the FI (*6*, *23*) calculated on the basis of the fraction of misplaced edges according to classical balance theory, i.e., the positive edges between communities and the negative edges within communities. We use two FIs in the main text. The first calculates the pairwise imbalance between communities, and the second calculates the overall imbalance of the partition. Given a pair of communities *i* and *j*, the pairwise FI is computed as

$${\text{FI}}_{\mathrm{ij}}=\frac{L_{\mathrm{ij}}^{+}+L_{\mathrm{ii}}^{-}+L_{\mathrm{jj}}^{-}}{L_{\mathrm{ii}}+L_{\mathrm{jj}}+L_{\mathrm{ij}}}$$ (4)

where $L_{\mathrm{ij}}^{+}$ represents the number of positive edges between community *i* and *j*, $L_{\mathrm{ii}}^{-}$ represents the number of negative edges within community *i*, $L_{\mathrm{ij}}$ represents the total number of edges between community *i* and community *j*, and $L_{\mathrm{ii}}$ represents the total number of edges within the community *i*.

Given a partition with *B* communities, the overall FI is defined as (*6*, *23*)

$$\text{FI}=\frac{\sum_{i=1}^{B}L_{\mathrm{ii}}^{-}+\sum_{i=1}^{B}\sum_{\left(>i\right)}L_{\mathrm{ij}}^{+}}{L}$$ (5)

where $\sum_{i=1}^{B}L_{\mathrm{ii}}^{-}$ represents the total number of negative edges within each community, $\sum_{i=1}^{B}\sum_{j\left(>i\right)}L_{\mathrm{ij}}^{+}$ the total number of positive edges between communities, and *L* the total number of edges overall. FI∈[0,1], with FI=1 indicating complete antibalance and FI=0 perfect balance.

### SBM inference

Here, we describe the statistical and computational procedures used in the methods based on SBMs, including the two SBM approaches we used in the survey (weighted and layered) and the corresponding hierarchical versions we used in case studies. The inference is performed using the Bayesian approach proposed in (*46*, *48*) and implemented in (*49*). In particular, the inference is nonparametric so that both the number of partitions *B* and the allocation of nodes to them are obtained simultaneously, following the principle of minimum description length (MDL) (*45*, *60*). For each network, we obtain 10 independent Markov chain Monte Carlo (MCMC) runs to characterize the posterior distribution of partitions and extract the representative partition that most samples are aligned with [see our code in (*61*) for details]. We perform our pairwise-classification of the relationship between groups using the consensus partition (*62*) obtained over these MCMC runs. By aggregating the outcomes over all plausible partitions, we focus on the most stable and statistically significant mesoscale organization of the network.

To evaluate the robustness of our mesoscale classification over alternative partitions, we quantify the variation of our classification over 1000 individual partitions sampled from the posterior distribution of the SBM model. These partitions were obtained by sampling 100 uncorrelated partitions (after equilibration) in each of the 10 independent MCMC runs. For each sampled partition, we extracted the dominant mesoscale type across all block pairs, recorded it as the sample winner, and compared it to the dominant mesoscale type obtained in the consensus partition. The certainty of the inferred dominant type was then quantified as the proportion of samples in which the dominant type prevailed, computed as

$$P=\frac{1}{k}\sum_{1}^{k}\delta \left(T^{\left(k\right)},T^{*}\right)$$ (6)

where $T^{\left(k\right)}$ is the dominant type extracted from the kth posterior sample, $T^{*}$ is the winner across all *k* samples, and δ(a,b)=1 if a=b and δ(a,b)=0 if a≠b. A high certainty, with agreement with the result in the consensus partition, indicates that the posterior is concentrated around one structure, meaning that the network has a robust and unambiguous community organization. A low certainty suggests that the posterior is diffuse across multiple structures, reflecting an ambiguity in the network’s organization.

To quantify the uncertainty in the number of blocks *B* in the partition, we use the effective number of blocks $B_{e}$. Unlike the discrete count *B*, $B_{e}$ is a continuous value that weights groups by their relative size, making it more sensitive to posterior fluctuations and less prone to zero-variance artifacts in averages. It is defined using the Shannon entropy of the group distribution

$$B_{e}=\text{exp}\left(-\sum_{r=1}^{B}p_{r}\text{ln}p_{r}\right)$$ (7)

where the weight $p_{r}=N_{r}/N$ is the fraction of nodes in group *r*. Note that $B_{e}=B$ only when all groups are of equal size; otherwise, $B_{e}<B$.

The results obtained for all networks in our survey and the two SBM methods are shown in the tables S3 and S4 and figs. S5 and S6. The results obtained for the four networks used in our case studies are reported in the tables S5 to S8. This analysis complements our bootstrap analysis (see Materials and Methods section “Classification robustness”), which validates the robustness of the block assignments themselves, while the posterior sampling described here specifically measures the stability of our classification over different (plausible) partitions of the network.

### Classification robustness

Our community structure classification is computed in Eq. 2 for a given network partition and using the density matrices $w^{\pm }$ defined in Eq. 1. To evaluate the statistical confidence of this classification for a pair of groups *r* and *s*, we adopt a bootstrapping method to estimate the robustness of the classification under small perturbations of the community structure while preserving the underlying density characteristics (*37*). Given a signed graph *G* (with $L^{+}$ positive and $L^{-}$ negative edges) and partition, we compute new positive and negative density matrices, $w^{\prime +}$ and $w^{\prime -}$, using a bootstrapping approach, i.e., randomly choosing $L^{+}$ and $L^{-}$ edges with replacement from the positive and negative edges (respectively) in *G*. We repeat the process *k* times and compute the certainty $R_{\mathrm{rs}}$ for each pair of communities *r* and *s* as the fraction of all *k* classifications $M_{\mathrm{rs}}^{\prime }$ that are equal to the original type $M_{\mathrm{rs}}$ as

$$R_{\mathrm{rs}}=\frac{1}{k}\sum_{1}^{k}\delta \left(M_{\mathrm{rs}},M_{\mathrm{rs}}^{\prime }\right)$$ (8)

where $M_{\mathrm{rs}}$ is the pairwise classification obtained from Eq. 2 and δ(a,b)=1 if a=b and δ(a,b)=0 if a≠b. This classification robustness score is the certainty for each pair of communities *r* and *s*, calculated as the fraction of trials that maintain the original classification.

### Normalized mutual information

The normalized mutual information (NMI) is a widely used metric to measure the similarity of detected communities (*63*, *64*). Given a network with *N* nodes, the partition $p=\left\{p_{1},\ldots ,p_{B}\right\}$ with *B* communities, and the ground truth $u=\left\{u_{1},\ldots ,u_{A}\right\}$ with *A* groups, we calculate the NMI as

$$\text{NMI}=\frac{2\sum_{i=1}^{A}\sum_{j=1}^{B}\frac{|p_{i}\cap u_{j}|}{N}\text{log}\left(\frac{\frac{|p_{i}\cap u_{j}|}{N}}{\frac{|p_{i}|}{N}\cdot \frac{|u_{j}|}{N}}\right)}{-\sum_{i=1}^{A}\frac{|p_{i}|}{N}\text{log}\frac{|p_{i}|}{N}-\sum_{j=1}^{B}\frac{|u_{j}|}{N}\text{log}\frac{|u_{j}|}{N}}$$ (9)

which corresponds to the mutual information between *p* and *u*, normalized by the combined entropy. NMI∈[0,1], with NMI=1 indicating perfect alignment and NMI=0 no alignment (zero mutual information).

### Model selection

Here, we describe an MDL model selection framework that quantifies the information gained from combining edge signs in the SBM description of the signed network. We compare our signed SBM model against an alternative null model in which the network’s mesoscale connectivity and the edge signs are strictly independent. Rather than assuming that signs universally improve clustering, our MDL framework explicitly measures whether the empirical sign distribution provides a compressible, structurally aligned signal. First, we fit the signed model using WSBM by passing edge signs as a discrete-binomial covariate (assuming that communities dictate both topology and signs) and extract its entropy $\Sigma_{\text{signed}}$ (*45*). The null model’s description length is

$$\Sigma_{\text{null}}=S\left(A^{\prime },b\right)+S\left(W\right)$$ (10)

where $S\left(A^{\prime },b\right)$ represents the entropy (compression) of the network using a standard, unsigned SBM and *S(W)* is the description length of the signs. In the computation of *S*(*W*), we consider the signs to be independent, identically distributed random variables governed solely by the network’s overall sign imbalance, irrespective of community structure. This is analytically defined as the negative log-marginal likelihood of the network’s signs

$$S\left(W\right)=-\text{ln}P\left(W\right)=\text{ln}\left(\begin{array}{c} L\\ L^{+} \end{array}\right)+\text{ln}\left(L+1\right)$$ (11)

where $L=L^{+}+L^{-}$ is the total number of edges and $L^{+}$ is the number of positive edges (see text S3 for the derivation). Last, by comparing these two models, any instance where our signed SBM yields a lower description length than the null hypothesis ($\Delta \Sigma =\Sigma_{\text{null}}-\Sigma_{\text{signed}}>0$) provides evidence that the edge signs are not merely global noise but are fundamentally informative of the network’s mesoscale structure.

### Balanced triads

To measure and compare the DoB (*4*, *14*, *22*, *26*, *27*) of the empirical networks here, we calculate the fraction of (weakly) balanced triads, which includes triads with all positive edges $T_{+++}$, all negative edges $T_{---}$, and two negative edges $T_{+--}$. The fraction of triads is calculated by adjacency matrix $A=A^{+}+A^{-}$ ($A^{+}\equiv {\left\{A_{\mathrm{ij}}^{+}\right\}}^{N}$, $A^{-}\equiv {\left\{A_{\mathrm{ij}}^{-}\right\}}^{N}$):

$$\begin{array}{c} T_{+++}=\frac{\text{Tr}\left[{\left(A^{+}\right)}^{3}\right]}{\text{Tr}\left[{\left(A^{+}-A^{-}\right)}^{3}\right]}\\ T_{---}=\frac{\text{Tr}\left[{\left(-A^{-}\right)}^{3}\right]}{\text{Tr}\left[{\left(A^{+}-A^{-}\right)}^{3}\right]}\\ T_{+--}=\frac{3\text{Tr}\left[A^{+}{\left(-A^{-}\right)}^{2}\right]}{\text{Tr}\left[{\left(A^{+}-A^{-}\right)}^{3}\right]}\\ T_{++-}=\frac{3\text{Tr}\left[{\left(A^{+}\right)}^{2}\left(-A^{-}\right)\right]}{\text{Tr}\left[{\left(A^{+}-A^{-}\right)}^{3}\right]} \end{array}$$ (12)

The DoB is then defined as the fraction of balanced triads $\text{DoB}=T_{+++}+T_{+--}$, and the weak DoB (WDoB) is then $\text{WDoB}=T_{+++}+T_{+--}+T_{---}$.

### Null-model testing

To determine whether observed network patterns are statistically significant or simply a byproduct of indirect network constraints, we use two distinct null-model testing frameworks. For our main analysis in the section “Micro and mesoscales exhibit different balance,” we use a mesoscale null model based on the SBM. This model allows us to isolate the effects of local structure from broader mesoscale organization. By sampling random networks from the inferred SBM (using either WSBM or LSBM), we generate a baseline that preserves the underlying block structure and the specific edge-sign densities within and between blocks. This approach ensures that the observed patterns in our results are not merely a reflection of the network’s community organization but are driven by specific social dynamics. The *z* score derived from this model suggests the primary findings presented in this study.

For the characterization of empirical networks (summarized in table S2), we use a sign-randomization null model. Since networks often contain an unequal fraction of positive and negative edges, raw counts alone are insufficient to quantify balance (*5*, *65*). Following (*27*), we create a control ensemble by fixing the underlying network topology and randomly shuffling the edge signs. This preserves the global sign density while removing any specific structural correlations.

To quantify the deviations of our observed patterns from the null-model expectations, we calculate the *z* score (*59*)

$$z\left(m\right)=\frac{N\left(m\right)-\langle N\left(m\right)\rangle }{\sigma \left(m\right)}$$ (13)

where *N*(*m*) is the number of pattern *m* (different kinds of triads) that appears in the empirical network, 〈N(m)〉 is the expected number of the same pattern under the null model, and $\sigma \left(m\right)=\sqrt{\langle N^{2}\left(m\right)\rangle -{\langle N\left(m\right)\rangle }^{2}}$ is the SD of *N(m)* under the null model. We adopt the convention where z(m)>2 indicates that the pattern *m* is overrepresented, meaning that it occurs significantly more than in the null model, and z(m)<−2 indicates that the pattern *m* is underrepresented, meaning that it occurs significantly less than in the null model.

## Supplementary Materials

### This PDF file includes:

Supplementary Text S1 to S3 Figs. S1 to S8 Tables S1 to S8 References
