# Supplementary material for: Mesoscale structures in signed networks
Source: Sci Adv. 2026 Oct 9;12(41):eaee5886. doi: 10.1126/sciadv.aee5886 (PMC13651842; doi:10.1126/sciadv.aee5886)
Supplement: Supplementary file 1 [file sciadv.aee5886_sm.pdf]

Science Advances
AAAS

Supplementary Materials for

Mesoscale structures in signed networks

Wei Zhang *et al.*

Corresponding author: Wei Zhang, wei.zhang4@sydney.edu.au

Sci. Adv. **12**, eaee5886 (2026)
DOI: 10.1126/sciadv.aee5886

This PDF file includes:

Supplementary Text S1 to S3
Figs. S1 to S8
Tables S1 to S8
References

S1 Data description

We considered three kinds of datasets: social-political networks, financial networks, and gene-regulatory networks. Although some networks originate from similar contexts, they vary substantially in structural characteristics such as size, edge density, and the ratio of negative to positive edges. These differences allow us to capture heterogeneity within the same domain and test the robustness of our method across diverse network configurations.

There are 18 social-political networks. *New Guinea Tribes* (66) reflected political alliances and oppositions among 16 tribes, developed through fieldwork involving observations and conversations with leaders and members in the Central Highlands. *Fraternity* (67) was created from a 15-week survey of 17 fraternity brothers at the University of Michigan in 1956, representing relationships with positive or negative edge values. *Monastery* (68) was collected via a questionnaire asking each monk to list "3 brothers who you like the most and the least" among 18 monks at an American monastery. *College House A, B, C* (69) come from a small group survey on evaluations of other group members (sorority sisters) based on a range of behavioral characteristics. *Swiss Student* (70) collected information about the relationships among Swiss students who shared most of their classes and often did coursework in groups. *Spanish High School A, B, C* (2) gathers data on student relationships by having students name their connections and rate them from -2 to $+2$. *Philosophers* (71) compiled master-pupil and acquaintanceship ties among philosophers from 800 B.C.E to 1935 C.E., based on historical texts in Randall Collins' seminal book. *Wiki Conflict* (72) represents conflicts between English Wikipedia users, where nodes represent users and edges indicate positive or negative interactions. A negative interaction occurs when one user reverts another's edit. *U.S. Congress* (52) illustrates bill co-sponsorship tendencies in the House and Senate. This signed network is derived from a bipartite network using the Stochastic Degree Sequence Model (52). Positive and negative edges indicate significant tendencies to co-sponsor or not. *Correlates of Wars* (53) offers data on international political relationships from 1946 to 1997, updated every four years. Positive edges indicate alliances or political agreements, while negative edges signify enmities or disagreements. *Twitter Referendum* (1) is an undirected signed network capturing Twitter user sentiments on the 2016 Italian Referendum, where edges are weighted -1 for opposing stances and $+1$ for the same stance. *Signed Citation network* (73) consists of papers from the field of Computational Linguistics. The positive (endorsement), negative (criticism), and neutral citations are classified by keyword-based citation sentiment. Following (74), we combine neutral and positive citations as positive, since authors do not express clear opposing views in neutral citations. *TwitterSG* (3) is a signed network drawn from Twitter interactions between 20th May and 8th August 2021. Here, we select the popular topics of the Australian Football League and the National Rugby League. A positive connection exists from user A to user B if user A likes a tweet posted by user B. Conversely, a negative connection exists from user A to user B if user A expresses opposition to user B's tweet in a reply.

In gene-regulatory networks (75), *Yeast* and *Escherichia coli* are classified as transcriptional networks, while the networks involving the Epidermal Growth Factor Receptor (*EGFR*) and *Macrophage* are signaling networks. The *Yeast* network relates to Saccharomyces cerevisiae, and the *Escherichia coli* network pertains to Escherichia coli. *Macrophage* are white blood cells that eliminate harmful substances like cancer cells, while *EGFR* is linked to a protein that promotes cell division and survival in epidermal tissues.

The financial networks consist of Bitcoin traders, with *Bitcoin OTC* and *Bitcoin Alpha* (76) built on user ratings of trust on trading platforms, ranging from -10 for fraudsters to $+10$ for complete trust. The decision to symmetrize directed networks was primarily driven by the need for a fair, cross-method comparison, as baseline methods like Louvain and Spin-glass are designed for undirected graphs.

Table S1 Dataset of directed empirical networks.
The table shows the total number of nodes N, the total number of edges L, the ratio of negative and positive edges L^-/L^+, and counts (with percentages) of +/– unidirectional, +/– reciprocated, conflicting links, and self loops. The percentages represent the relative frequency of each link type as a proportion of the total number of links in the network.

Name	N	L	$\frac{L^-}{L^+}$	$\xrightarrow{+}$ (%)	$\xrightarrow{-}$ (%)	$\overset{+}{\leftrightarrow}$ (%)	$\overset{-}{\leftrightarrow}$ (%)	conflicting (%)	self loop (%)
Monastery (68)	18	103	0.84	25 (24%)	28 (27%)	30 (29%)	18 (17%)	2 (2%)	0 (0%)
College House A (69)	21	94	0.84	24 (26%)	36 (38%)	26 (28%)	6 (6%)	2 (2%)	0 (0%)

Table S1 continued from previous page

Name	N	L	$\frac{L^-}{L^+}$	$\xrightarrow{+}$ (%)	$\xrightarrow{-}$ (%)	$\overset{+}{\leftrightarrow}$ (%)	$\overset{-}{\leftrightarrow}$ (%)	conflicting (%)	self loop (%)
College House B (69)	17	83	1.02	15 (18%)	30 (36%)	24 (29%)	10 (12%)	4 (5%)	0 (0%)
College House C (69)	20	81	1	22 (28%)	26 (32%)	18 (22%)	14 (18%)	0 (0%)	1 (1%)
Swiss Studentlife (70)	181	713	0.21	278 (39%)	108 (15%)	307 (43%)	10 (1%)	10 (1%)	0 (0%)
Spanish High School A (2)	409	8,557	0.17	3,425 (40%)	1,036 (12%)	3,780 (44%)	122 (1%)	194 (2%)	0 (0%)
Spanish High School B (2)	238	3,755	0.11	1,755 (47%)	268 (7%)	1,580 (42%)	28 (1%)	124 (3%)	0 (0%)
Spanish High School C (2)	534	12,812	0.16	4,843 (38%)	1,399 (11%)	5,992 (47%)	200 (2%)	378 (3%)	0 (0%)
Citations (73)	8,711	25,161	0.09	22,910 (91%)	2,157 (9%)	57 (0%)	0 (0%)	14 (0%)	23 (0%)
TwitterSG (3)	3,883	6,338	0.91	3,290 (52%)	2,832 (45%)	17 (0%)	182 (3%)	17 (0%)	0 (0%)
Yeast (75)	664	1,064	0.26	844 (79%)	220 (21%)	0 (0%)	0 (0%)	0 (0%)	0 (0%)
Escherichia coli (75)	1,376	3,150	0.70	931 (67%)	466 (33%)	0 (0%)	0 (0%)	0 (0%)	0 (0%)
EGFR (75)	313	755	0.50	499 (66%)	256 (34%)	220 (21%)	0 (0%)	0 (0%)	0 (0%)
Macrophage (75)	660	1,397	0.50	931 (67%)	466 (33%)	0 (0%)	0 (0%)	0 (0%)	0 (0%)
Bitcoin OTC, 2015 (76)	348	998	0.09	269 (27%)	63 (6%)	646 (65%)	12 (1%)	8 (1%)	0 (0%)
Bitcoin Alpha, 2014 (76)	687	2,714	0.18	660 (24%)	332 (12%)	1,618 (60%)	62 (2%)	42 (2%)	0 (0%)

The statistics and basic description of the dataset after preprocessing are summed up in Table S2. Many of the empirical networks here are characterized by a small density $c = 2L/N(N-1)$ and a small fraction of negative links L^-/L^+.

Our dataset consists primarily of social networks, reflecting the availability of signed network data in this domain. To assess whether this overrepresentation affects our findings, we examined within each domain separately (Figs. S1-S3) and found that the result, that unbalanced structures are common, was qualitatively similar across domains. This suggests that the observed patterns are not driven solely by social networks but are consistent across different contexts.

Table S2 Descriptive statistics of the largest component of empirical networks.
The table shows the total number of nodes N, the total number of edges L, the ratio of negative and positive edges L^-/L^+, the average degree $\langle k \rangle$, the network density $c = 2L/N(N-1)$, and T_{+++}, T_{++-}, T_{+--} and T_{---} are the fractions of 3-cycles (triads) with all positive edges, one negative edge, two negative edges, and all negative edges, respectively. Their corresponding z-scores are shown in parentheses (see Methods 4.7). DoB is the fraction of balanced triads, that is, the fraction of triads with even negative edges. Most empirical networks exhibit strong negative z-scores for triads with two positive edges. This indicates that unbalanced triads are underrepresented. These findings provide strong support for classical balance theory, as this "forbidden" configuration is notably avoided. For social-political networks, the results consistently show positive z-scores for most positive triads, but mixed z-scores for the "enemy's enemy" triad $(+--)$ and the "all enemies" triad $(---)$. This suggests that some networks strongly adhere to the balance principle, while others do not. For example, the *Spanish High School A, B, C* shows a significant overrepresentation of the $(---)$ configuration, which supports the weak balance theory. Gene-regulatory networks tend to suppress structural balance, avoiding both clusters of positive relationships $(+++)$ and "enemy's enemy" configurations $(+--)$. Besides, transcriptional networks (*Yeast* and *Escherichia coli*) generally avoid unbalanced $(++-)$ configurations to maintain hierarchical control, signaling networks (*EGFR* and *Macrophage*) appear to favor them. Financial networks show weaker balance effects overall, suggesting different underlying mechanisms than pure social balance theory.

Name	N	L	$\frac{L^-}{L^+}$	$\langle k \rangle$	c	T_{+++} (z-score)	T_{++-} (z-score)	T_{+--} (z-score)	T_{---} (z-score)	DoB
New Guinea Tribes (66)	16	58	1	7.25	0.48	0.279 (4.48)	0.029 (-6.28)	0.588 (3.70)	0.103 (-0.42)	0.867
Fraternity (67)	17	40	0.74	4.71	0.29	0.556 (4.51)	0 (-3.82)	0.222 (-0.89)	0.222 (2.73)	0.778
Monastery (68)	18	78	0.95	8.67	0.51	0.182 (1.89)	0.122 (-5.88)	0.583 (4.87)	0.113 (0.025)	0.765
College House A (69)	21	77	1.08	7.33	0.37	0.229 (4.08)	0.133 (-4.54)	0.482 (1.77)	0.157 (0.60)	0.711
College House B (69)	17	64	1.37	7.53	0.47	0.065 (-0.20)	0.221 (-1.85)	0.481 (0.91)	0.234 (1.23)	0.546
College House C (69)	20	65	1.03	6.50	0.34	0.211 (2.0)	0.038 (-4.93)	0.673 (4.63)	0.077 (-1.27)	0.884
Swiss Studentlife (70)	181	546	0.28	6.03	0.034	0.689 (6.73)	0.15 (-9.15)	0.15 (2.22)	0.011 (0.26)	0.839
Spanish High School A (2)	409	6,509	0.22	31.83	0.078	0.629 (16.36)	0.182 (-51.20)	0.169 (37.41)	0.020 (29.89)	0.798
Spanish High School B (2)	238	2,889	0.14	24.28	0.102	0.729 (5.84)	0.183 (-15.15)	0.082 (18.75)	0.005 (9.71)	0.811
Spanish High School C (2)	534	9,527	0.22	35.68	0.033	0.614 (14.67)	0.231 (-46.91)	0.138 (36.54)	0.017 (34.37)	0.752
Philosophers (71)	218	259	0.126	2.38	0.011	0.75 (0.38)	0.083 (-1.42)	0.167 (2.49)	0 (-0.14)	0.917
Wiki Conflict, 2009 (72)	359	2,535	1.33	14.12	0.039	0.088 (0.36)	0.109 (-5.46)	0.675 (11.53)	0.128 (-1.41)	0.763
U.S. Congress, H100 (52)	446	50,688	2.64	227	0.51	0.102 (460.54)	0.018 (-210.88)	0.492 (141.77)	0.388 (5.82)	0.594
U.S. Congress, S100 (52)	101	2,143	2.52	42.44	0.42	0.116 (68.99)	0.02 (-34.48)	0.387 (-13.05)	0.478 (17.19)	0.503
Correlates of Wars 1954-57 (53)	80	492	0.178	12.3	0.16	0.869 (14.06)	0.093 (-16.91)	0.03 (-3.84)	0.008 (3.81)	0.899
Twitter Referendum (1)	10,864	251,396	0.054	46.28	0.004	0.938 (48.21)	0.027 (-71.41)	0.035 (150.56)	0 (-15.91)	0.973
Citations (73)	8,711	25,073	0.094	5.76	0.001	0.779 (1.32)	0.179 (-3.54)	0.039 (6.67)	0.003 (3.41)	0.818
TwitterSG (3)	3,883	5,480	0.952	2.82	0.001	0.495 (14.37)	0.292 (-2.90)	0.135 (-7.39)	0.078 (-1.63)	0.63
Yeast (75)	664	1,064	0.26	3.20	0.005	0.486 (-0.13)	0.371 (-0.33)	0.057 (-0.95)	0.086 (6.91)	0.543
Escherichia coli (75)	1,376	3,150	0.705	4.58	0.003	0.251 (1.23)	0.374 (-2.49)	0.248 (-1.43)	0.127 (2.76)	0.499

Table S2 continued from previous page

Name	N	L	$\frac{L^-}{L^+}$	$\langle k \rangle$	c	T_{+++} (z-score)	T_{++-} (z-score)	T_{+--} (z-score)	T_{---} (z-score)	DoB
EGFR (75)	313	755	0.51	4.82	0.015	0.028 (-10.42)	0.873 (17.09)	0.034 (-8.96)	0.064 (2.60)	0.062
Macrophage (75)	660	1,397	0.50	4.23	0.006	0.024 (-14.63)	0.806 (21.29)	0.015 (-12.62)	0.155 (16.55)	0.039
Bitcoin OTC, 2015 (76)	348	665	0.12	3.82	0.011	0.908 (4.69)	0.063 (-5.38)	0.029 (-0.19)	0 (-0.56)	0.937
Bitcoin Alpha, 2014 (76)	687	1,853	0.26	5.39	0.008	0.747 (9.61)	0.12 (-17.36)	0.131 (2.31)	0.002 (-2.71)	0.878

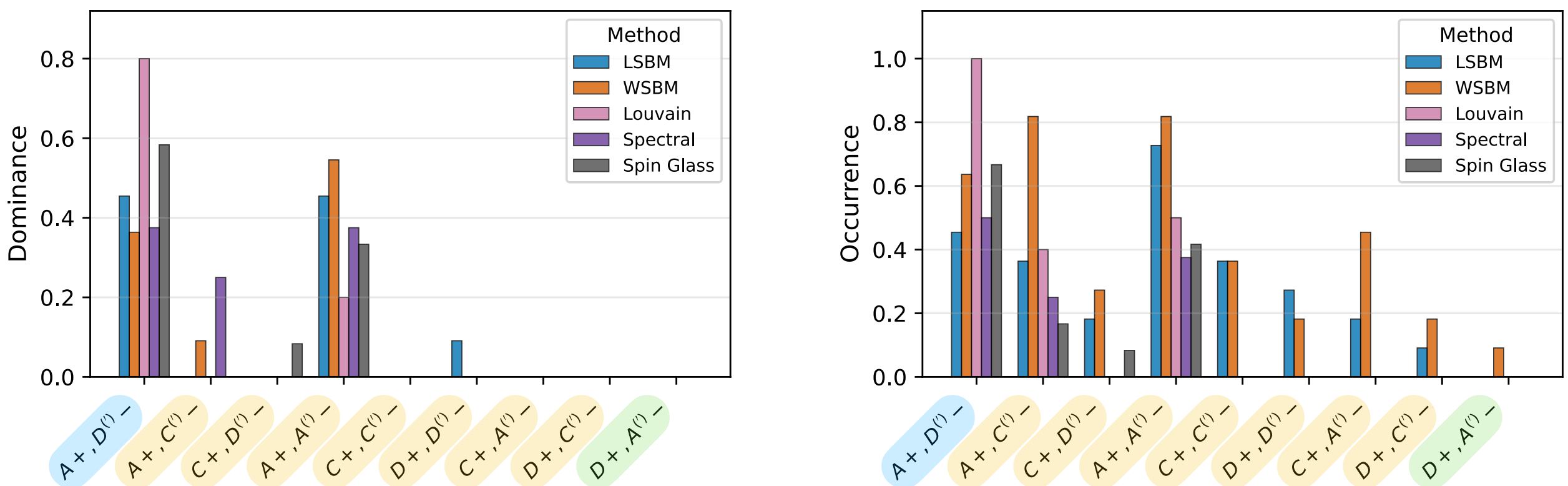

Figure S1: **Community types in social-political networks.** Left: fraction of networks in which each community type is dominant. Right: fraction of networks in which each community type occurs.

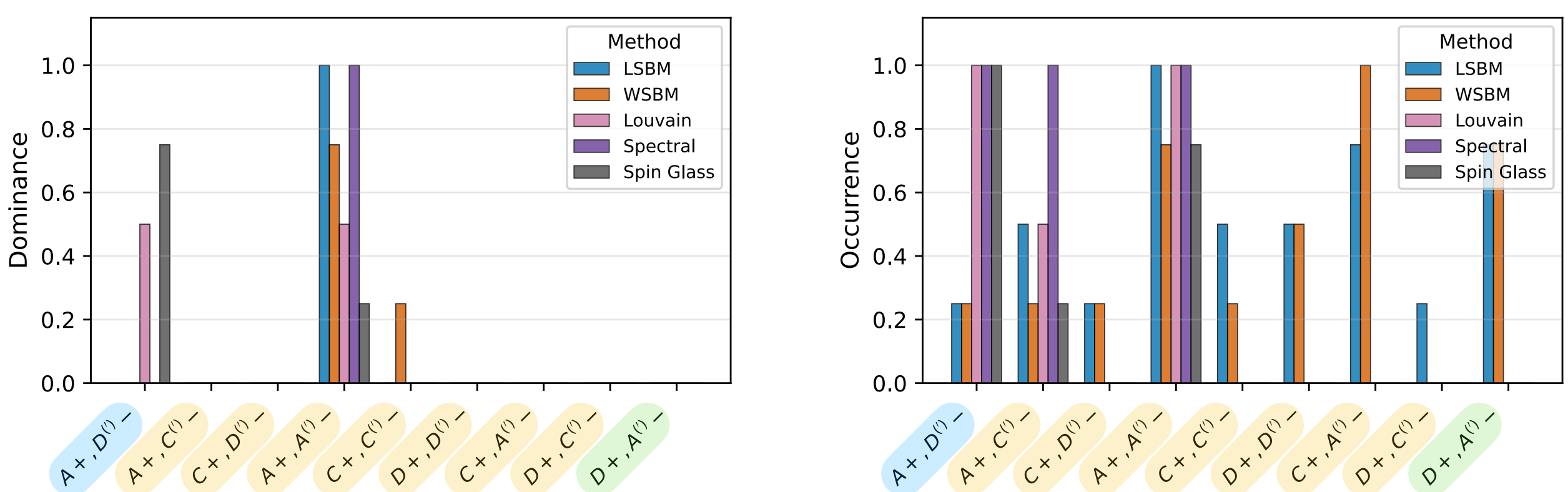

Figure S2: **Community types in gene-regulatory networks.** Left: fraction of networks in which each community type is dominant. Right: fraction of networks in which each community type occurs.

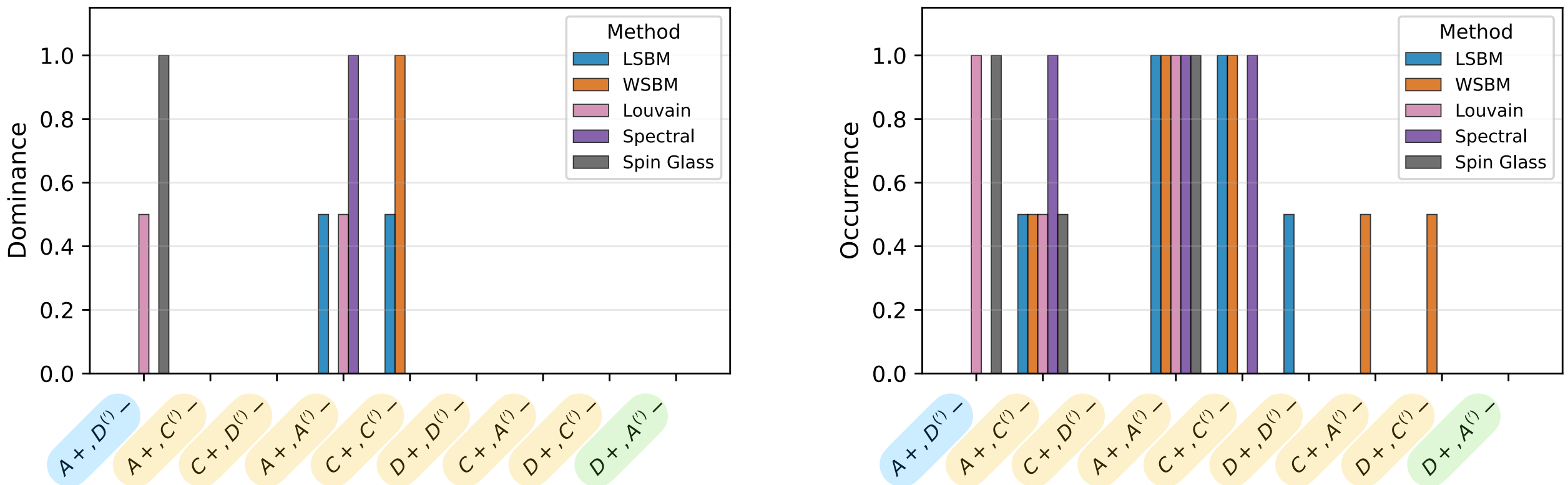

Figure S3: **Community types in financial networks.** Left: fraction of networks in which each community type is dominant. Right: fraction of networks in which each community type occurs.

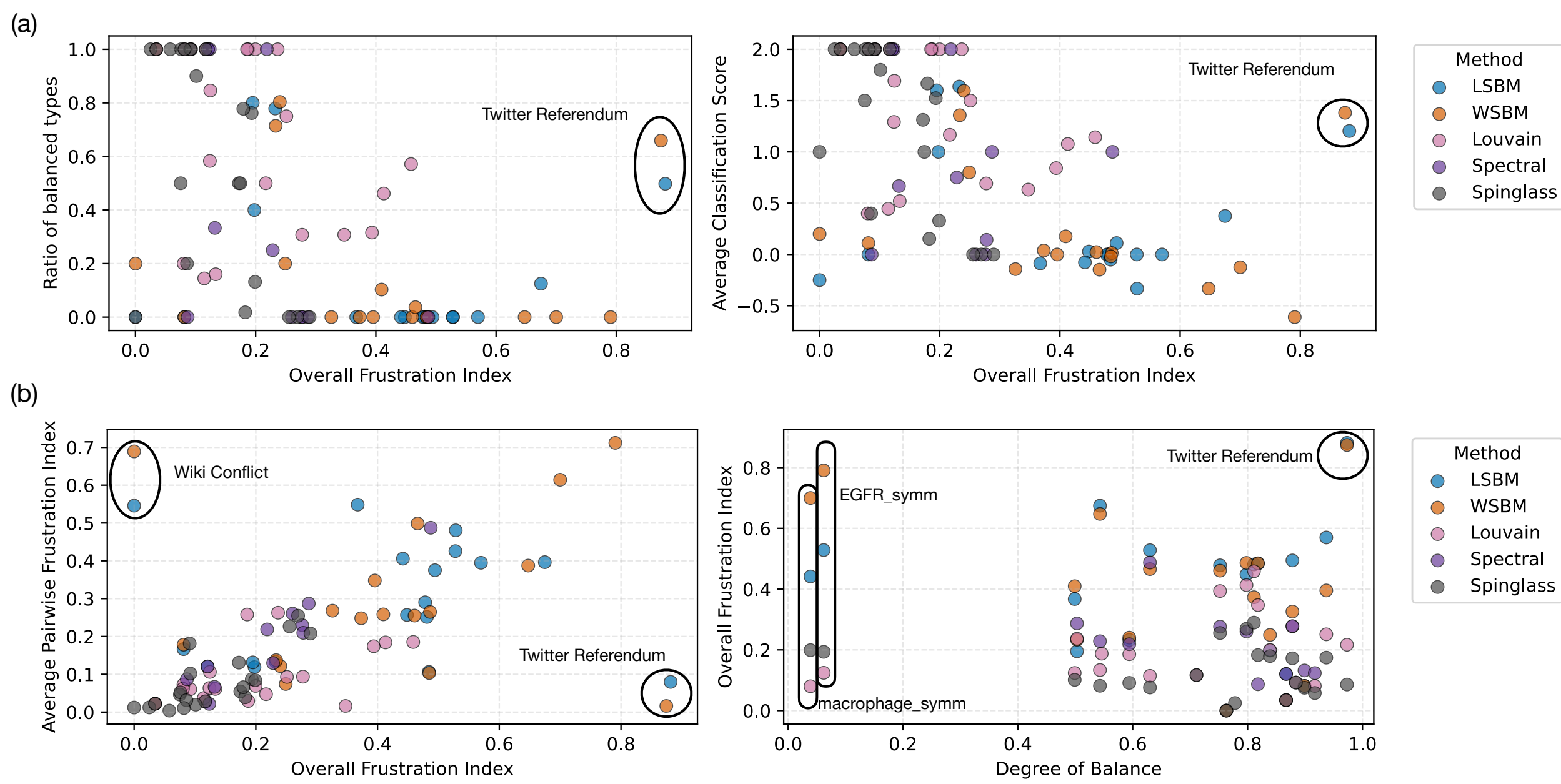

Figure S4: **Other relationships between meso-scale (im)balance and micro-scale balance.** (a) Relationship between structure classifications and the overall FI given the partition of the network. Classification of balanced structure types (left panel), and average balance score (right panel), with the prediction from traditional balance theory, qualified by the overall frustration index. The results show that our classification closely matches theoretical structural balance expectations. (b) Relationship between average pairwise FI, overall FI, and the ratio of balanced triads. The left panel confirms that the average pairwise frustration index could reflect the overall balance degree of the partition. In addition, the SBMs could find more unbalanced communities compared with other methods. The right panel shows that the ratio of balanced triads from a microscopic perspective could not reflect the balance degree of a given partition at a mesoscopic level.

S2 Implementation of SBMs

The comparison of consensus partition and the sampling from the posterior distribution are shown in the Table S3, Table S4, and Figure S5. The posterior uncertainty analysis follows Methods 4.3. The posterior sampling initializes the state with a partition into one group. After we equilibrate the Markov chain following the principle of minimum description length, we collect partitions. The details implemented in Ref. (49). For most networks, there is almost the same between the classification obtained in the consensus partition and through the posterior sampling. The dominant type identified in the consensus partition remains the dominant type in the sampling analysis (i.e., 96% consistency for LSBM and 92% for WSBM). For the outliers, although the dominant type in the consensus differs from that in the sampling analysis, we found that this type is the second most common and occurs at a frequency very close to the dominant type. Unbalanced structures were predominant, with 58% of networks for LSBM and 67% for WSBM. Specifically, the $(A^+, A^{(')-})$ configuration emerged as the most stable structure, particularly in large networks where the structural signal is strongest.

Table S3 Details of the inferred structures from posterior sampling by LSBM.
The table shows the consensus partition results (left) and posterior sampling analysis (right) by LSBM as a comparison. For the consensus results, we report (i) the dominant type among the retained blocks, (ii) the average robustness $\langle R\rangle$ of that dominant type (see Methods 4.4), (iii) the number of blocks B after filtering out small communities (those with fewer than $\min([0.05N, 5])$ nodes are discarded), (iv) the effective number of blocks B_e, and (v) the frustration index FI (see Methods 4.2). For the posterior sampling (Methods 4.3), we report: (i) the dominant type, representing the most frequent interaction type observed across the samples, (ii) the Certainty Score P, defined as the proportion of MCMC samples where the dominant type remained consistent, (iii) the average number of blocks ($B \pm$ std) after filtering out small communities (those with fewer than $\min([0.05N, 5])$ nodes are discarded), (iv) the average effective number of blocks ($B_e \pm$ std), and (v) the Frustration Index (FI $\pm$std) calculated over the samples. In *Bitcoin OTC 2015*, the consensus dominant type $(A^+,\ A^{(')-})$ (marked by †) differs from the the most frequent interaction type $(C^+,\ C^{(')-})$ obtained in the sampling, but appears as the second most frequent type with a frequency close to the dominant one. Because the posterior landscape is likely flat across multiple near-equivalent configurations.

Name	Consensus					Sampling				
	Dominant	$\langle R\rangle$	B	B_e	FI	Dominant	P	$\langle B\rangle$	B_e	FI
New Guinea Tribes	$(A+, D^{(')}-)$	0.99	2	1.75	0.12	$(A+, D^{(')}-)$	0.99	2.24 (45)	2.03 (51)	0.11 (5)
Fraternity	-	-	1	1.00	-	-	0.96	1.04 (18)	1.03 (17)	-
Monastery	-	-	1	1.00	-	-	0.96	1.03 (19)	1.03 (18)	-
College House A	-	-	1	1.00	-	-	0.63	1.45 (67)	1.41 (62)	-
College House B	-	-	1	1.00	-	-	0.87	1.13 (35)	1.11 (31)	-
College House C	$(A+, D^{(')}-)$	0.66	2	1.84	0.11	$(A+, D^{(')}-)$	0.98	1.99 (16)	1.84 (14)	0.11 (2)
Swiss Studentlife	$(A+, A^{(')}-)$	0.78	8	6.97	0.27	$(A+, A^{(')}-)$	0.99	6.96 (104)	6.65 (96)	0.28 (3)
Spanish High School A	$(A+, A^{(')}-)$	0.83	13	11.09	0.43	$(A+, A^{(')}-)$	1.00	13.15 (107)	11.30 (63)	0.44 (1)
Spanish High School B	$(A+, A^{(')}-)$	0.78	11	10.30	0.38	$(A+, A^{(')}-)$	1.00	11.75 (57)	10.87 (38)	0.43 (3)
Spanish High School C	$(A+, A^{(')}-)$	0.84	20	19.41	0.50	$(A+, A^{(')}-)$	1.00	20.28 (45)	19.75 (40)	0.50 (0)
Philosophers	-	-	1	1.00	-	-	0.99	1.00 (0)	1.00 (0)	-
Wiki Conflict 2009	$(C+, C^{(')}-)$	0.83	4	3.03	0.00	$(C+, C^{(')}-)$	0.86	3.90 (54)	2.84 (46)	0.00 (0)
US Congress H100	$(A+, D^{(')}-)$	0.88	22	20.85	0.24	$(A+, D^{(')}-)$	1.00	21.79 (61)	20.63 (54)	0.23 (0)
US Congress S100	$(A+, D^{(')}-)$	0.84	6	5.50	0.17	$(A+, D^{(')}-)$	1.00	6.13 (35)	5.77 (40)	0.19 (1)
CoW 1954-57	$(A+, A^{(')}-)$	0.77	5	4.75	0.07	$(A+, A^{(')}-)$	1.00	5.18 (39)	4.89 (29)	0.08 (4)
Twitter Referendum	$(A+, D^{(')}-)$	0.91	64	32.44	0.90	$(A+, D^{(')}-)$	1.00	63.18 (165)	36.52 (123)	0.88 (1)
Citations	$(A+, A^{(')}-)$	0.91	41	30.25	0.61	$(A+, A^{(')}-)$	1.00	41.52 (277)	33.21 (295)	0.62 (6)
Twitter SG	$(A+, A^{(')}-)$	0.97	5	2.69	0.55	$(A+, A^{(')}-)$	0.95	5.30 (46)	3.13 (29)	0.53 (2)

Table S3 continued from previous page

Name	Consensus					Sampling				
	Dominant	$\langle R\rangle$	B	B_e	FI	Dominant	P	$\langle B\rangle$	B_e	FI
Yeast	$(D+, D^{(\prime)}-)$	0.58	4	2.64	0.78	$(D+, D^{(\prime)}-)$	0.42	4.80 (87)	3.13 (50)	0.76 (1)
Escherichia coli	$(A+, A^{(\prime)}-)$	0.83	12	4.00	0.46	$(A+, A^{(\prime)}-)$	0.91	12.29 (140)	6.56 (121)	0.37 (8)
EGFR	$(A+, A^{(\prime)}-)$	0.84	6	5.69	0.52	$(A+, A^{(\prime)}-)$	1.00	6.84 (78)	6.15 (43)	0.53 (2)
Macrophage	$(A+, A^{(\prime)}-)$	0.94	12	10.51	0.56	$(A+, A^{(\prime)}-)$	0.99	11.10 (104)	9.89 (113)	0.64 (12)
Bitcoin OTC 2015	$(A+, A^{(\prime)}-)^{\dagger}$	0.77	5	3.18	0.62	$(C+, C^{(\prime)}-)$	0.48	4.70 (69)	3.40 (46)	0.61 (3)
Bitcoin Alpha 2014	$(C+, C^{(\prime)}-)$	0.90	8	3.79	0.54	$(C+, C^{(\prime)}-)$	0.94	7.76 (63)	4.34 (37)	0.54 (2)

Table S4 Details of the inferred structures from posterior sampling by WSBM.
The table shows the consensus partition results (left) and posterior sampling analysis (right) by WSBM as a comparison. For the consensus results, we report (i) the dominant type among the retained blocks, (ii) the average robustness $\langle R\rangle$ of that dominant type (see Methods 4.4), (iii) the number of blocks B after filtering out small communities (those with fewer than $\min([0.05N, 5])$ nodes are discarded), (iv) the effective number of blocks B_e, and (v) the frustration index FI (see Methods 4.2). For the posterior sampling (Methods 4.3), we report: (i) the dominant type, representing the most frequent interaction type observed across the samples, (ii) the Certainty Score P, defined as the proportion of MCMC samples where the dominant type remained consistent, (iii) the average number of blocks ($B \pm$ std) after filtering out small communities (those with fewer than $\min([0.05N, 5])$ nodes are discarded), (iv) the average effective number of blocks ($B_e \pm$ std), and (v) the Frustration Index (FI $\pm$std) calculated over the samples. For *Monastery* and *TwitterSG*, the consensus dominant type (marked by †) differs from the most frequent interaction type obtained via sampling. However, it is the second most frequent type and occurs at a frequency close to the sampling dominant type. Because the posterior landscape is likely flat across multiple near-equivalent configurations.

Name	Consensus					Sampling				
	Dominant	$\langle R\rangle$	B	B_e	FI	Dominant	P	$\langle B\rangle$	B_e	FI
New Guinea Tribes	$(A+, D^{(\prime)}-)$	1.00	3	2.92	0.03	$(A+, D^{(\prime)}-)$	1.00	3.01(13)	2.93(12)	0.04(02)
Fraternity	$(A+, C^{(\prime)}-)$	0.76	2	1.73	0.10	$(A+, C^{(\prime)}-)$	0.97	2.01(10)	1.74(11)	0.10(03)
Monastery	$(C+, C^{(\prime)}-)^{\dagger}$	0.43	3	2.46	0.24	$(A+, D^{(\prime)}-)$	0.81	3.39(69)	3.22(63)	0.27(08)
College House A	$(A+, D^{(\prime)}-)$	0.67	4	3.21	0.19	$(A+, D^{(\prime)}-)$	0.94	3.63(61)	3.28(50)	0.25(07)
College House B	$(A+, C^{(\prime)}-)$	0.53	2	1.91	0.27	$(A+, C^{(\prime)}-)$	0.40	2.33(48)	2.22(49)	0.32(11)
College House C	$(A+, D^{(\prime)}-)$	0.66	2	1.84	0.11	$(A+, D^{(\prime)}-)$	1.00	2.03(16)	1.87(18)	0.11(02)
Swiss Studentlife	$(A+, A^{(\prime)}-)$	0.69	7	6.78	0.46	$(A+, A^{(\prime)}-)$	0.81	7.29(65)	6.92(67)	0.47(04)
Spanish High School 1	$(A+, A^{(\prime)}-)$	0.82	16	14.59	0.48	$(A+, A^{(\prime)}-)$	1.00	18.14(85)	16.02(67)	0.53(02)
Spanish High School 2	$(A+, A^{(\prime)}-)$	0.73	12	11.17	0.42	$(A+, A^{(\prime)}-)$	1.00	12.46(56)	11.51(40)	0.45(02)
Spanish High School 3	$(A+, A^{(\prime)}-)$	0.85	24	22.14	0.48	$(A+, A^{(\prime)}-)$	1.00	24.75(68)	22.69(69)	0.49(01)
Philosophers	$(D+, A^{(\prime)}-)$	1.00	2	1.67	0.51	$(D+, A^{(\prime)}-)$	0.43	2.40(49)	2.17(39)	0.61(10)
Wiki Conflict 2009	$(A+, A^{(\prime)}-)$	0.96	7	4.95	0.00	$(A+, A^{(\prime)}-)$	1.00	6.62(50)	4.58(63)	0.00(00)
US Congress H100	$(A+, D^{(\prime)}-)$	0.93	30	28.54	0.25	$(A+, D^{(\prime)}-)$	1.00	29.50(67)	28.10(66)	0.25(00)
US Congress S100	$(A+, D^{(\prime)}-)$	0.75	8	7.82	0.18	$(A+, D^{(\prime)}-)$	1.00	8.21(41)	8.03(39)	0.18(01)
CoW 1954-57	$(A+, A^{(\prime)}-)$	0.94	5	4.72	0.09	$(A+, A^{(\prime)}-)$	1.00	5.01(07)	4.74(06)	0.09(01)
Twitter Referendum	$(A+, D^{(\prime)}-)$	0.98	46	26.37	0.88	$(A+, D^{(\prime)}-)$	1.00	47.60(191)	30.06(163)	0.88(00)
Citations	$(A+, A^{(\prime)}-)$	0.92	37	27.88	0.56	$(A+, A^{(\prime)}-)$	1.00	37.00(184)	29.33(194)	0.61(03)
Twitter SG	$(C+, A^{(\prime)}-)^{\dagger}$	0.70	13	6.82	0.47	$(A+, A^{(\prime)}-)$	0.43	12.35(188)	7.21(90)	0.49(03)
Yeast	$(A+, D^{(\prime)}-)$	0.64	7	4.15	0.76	$(A+, D^{(\prime)}-)$	0.76	6.90(30)	4.36(24)	0.76(02)

Table S4 continued from previous page

Name	Consensus					Sampling				
	Dominant	$\langle R \rangle$	B	B_e	FI	Dominant	P	$\langle B \rangle$	B_e	FI
Escherichia coli	$(A+, A^{(\prime)}-)$	0.75	16	7.72	0.30	$(A+, A^{(\prime)}-)$	0.95	17.84(142)	9.52(53)	0.38(10)
EGFR	$(D+, A^{(\prime)}-)$	0.90	7	6.74	0.92	$(D+, A^{(\prime)}-)$	0.95	7.99(149)	7.47(154)	0.92(02)
Macrophage	$(A+, A^{(\prime)}-)$	0.86	11	9.40	0.90	$(A+, A^{(\prime)}-)$	0.66	11.40(66)	10.06(61)	0.95(02)
Bitcoin OTC 2015	$(D+, A^{(\prime)}-)$	0.77	6	4.64	0.68	$(D+, A^{(\prime)}-)$	0.60	6.11(42)	4.86(46)	0.69(06)
Bitcoin Alpha 2014	$(A+, A^{(\prime)}-)$	0.91	12	7.03	0.54	$(A+, A^{(\prime)}-)$	0.81	11.44(115)	7.96(75)	0.56(03)

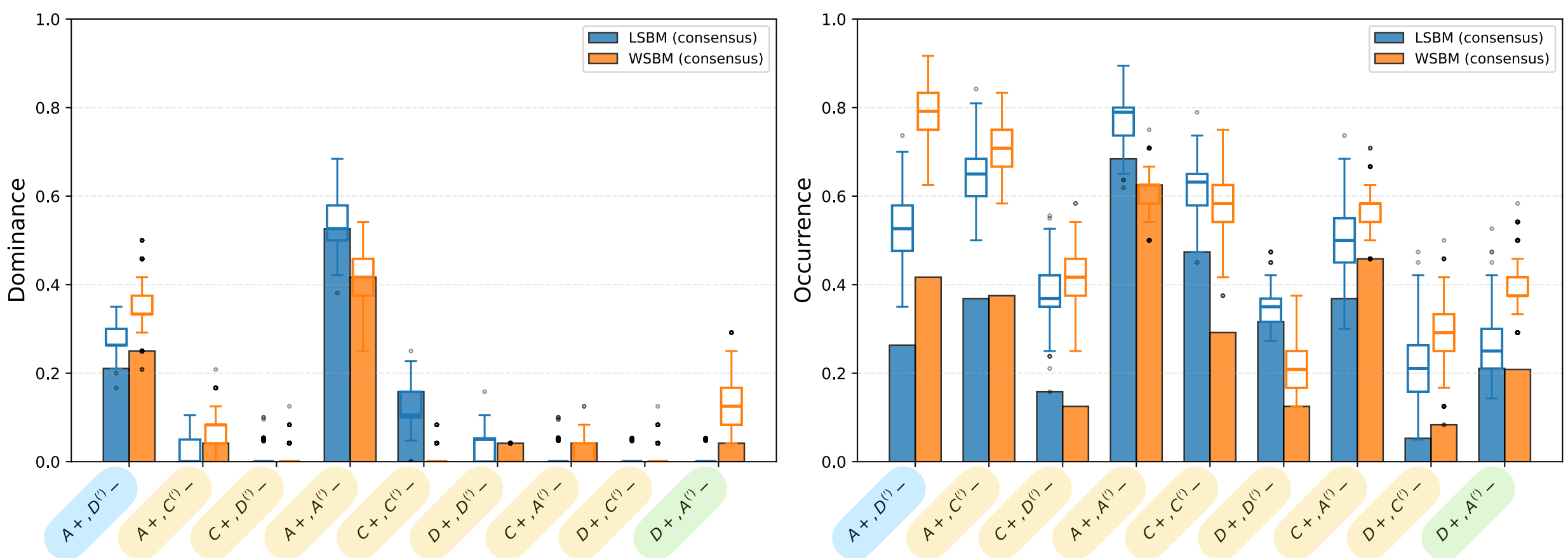

Figure S5: **Dominance and occurrence of structure types under consensus and sampling.** Bar heights represent the consensus estimate: the fraction of networks where each type is dominant (left panel) or occurs (right panel), derived from the consensus partition. Overlaid box plots summarize the distribution of the same quantities across 1,000 sampling runs. Dominance is defined as the fraction of networks where the type is the most frequent; occurrence is defined as the fraction of networks where the type appears at least once. Results are shown for both LSBM (blue) and WSBM (orange). The red horizontal line within each box indicates the median across samples. The agreement between the bar height and the box plot location indicates robustness of the consensus estimate to sampling variability.

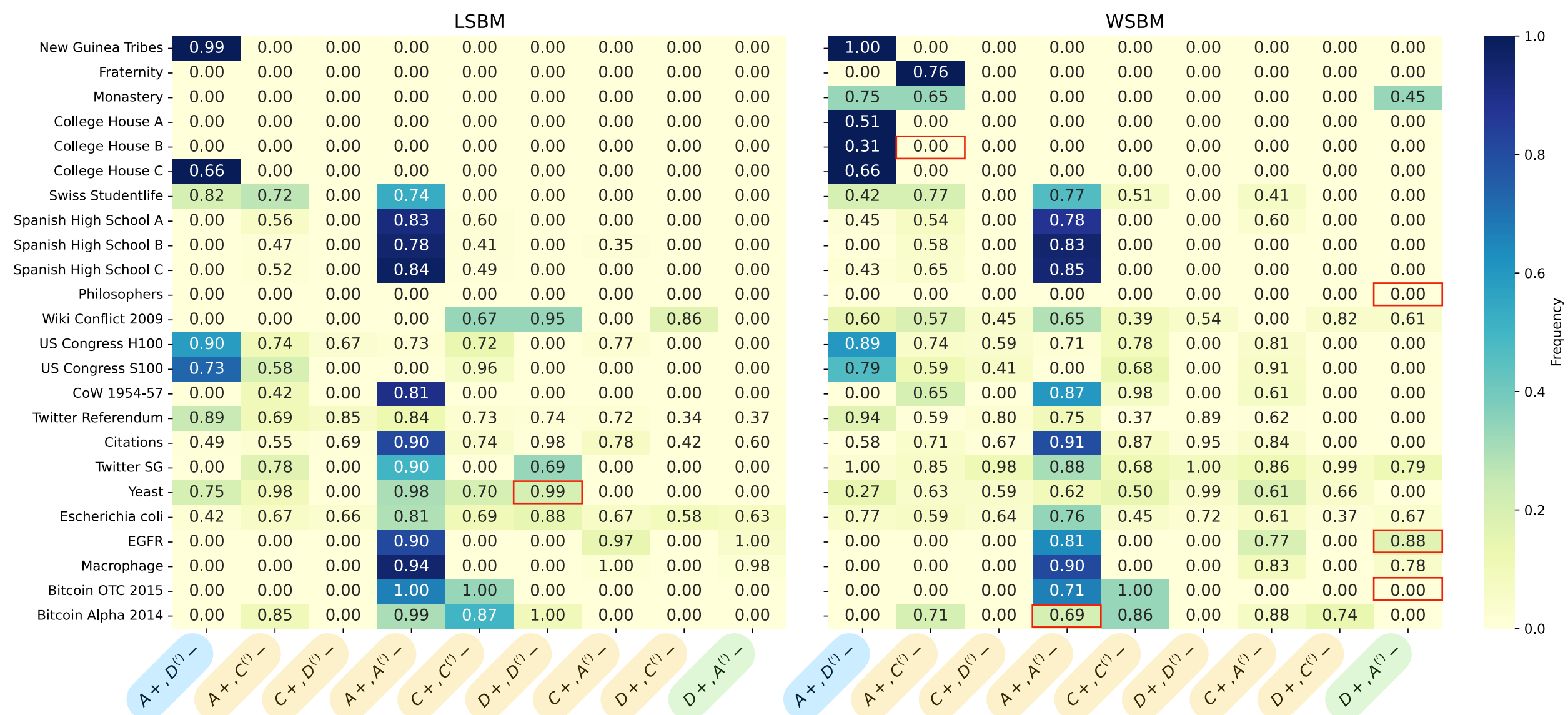

Figure S6: **Details of the inferred structures for the survey by LSBM and WSBM.** The heatmaps illustrate the frequency and stability of nine fundamental interaction types. The background color intensity represents the interaction frequency (normalized from 0.0 to 1.0), while the numerical annotations denote the mean robustness of the type. We compared the dominant types identified in our survey with those from the posterior samplings (Table S3-S4), and the inconsistent types were marked by red rectangles. For networks with a dominant-type certainty exceeding 0.5, the consistency rate was 100% for LSBM and 88% for WSBM. When considering all networks, the consistency rates were 96% for LSBM and 79% for WSBM. In cases of inconsistency, two main factors were identified. First, some discrepancies arise from low certainty in the posterior sampling (*Yeast* for LSBM, *College House B* and *Philosophers* for WSBM), which suggests there are alternative community structures. Second, in other cases (*ERFG*, *Bitcoin OTC 2015* and *Bitcoin Alpha 2014*), the survey-identified type corresponds to the second most frequent type in the posterior distribution, with a frequency very close to that of the dominant type.

Table S5: **Details of the inferred structures for the Spanish High School by nested WSBM.** The table shows hierarchical community structure results for consensus partition and posterior sampling (Methods 4.3). The consensus columns report the effective number of groups B_e, and the frequency of interaction types from the consensus partition. The sampling columns report the mean and standard deviation of the effective number of groups $\langle B_e \rangle$ and standard deviation, and the certainty of the interaction types. The dominant type of Level 1 is $(A^+, A^{(\prime)-})$, which confirms the results in Fig. 6.

Consensus										
Hierarchy	B_e	$(A^+, D^{(\prime)-})$	$(A^+, C^{(\prime)-})$	$(C^+, D^{(\prime)-})$	$(A^+, A^{(\prime)-})$	$(C^+, C^{(\prime)-})$	$(D^+, D^{(\prime)-})$	$(C^+, A^{(\prime)-})$	$(D^+, C^{(\prime)-})$	$(D^+, A^{(\prime)-})$
Level 0	9.73	0.04	0.07	0.0	0.86	0.02	0.0	0.02	0.0	0.0
Level 1	3.90	0.2	0.2	0.1	0.50	0.0	0.0	0.0	0.0	0.0
Level 2	1.90	0.0	0.0	0.0	1.0	0.0	0.0	0.0	0.0	0.0
Sampling										
Hierarchy	$\langle B_e \rangle$	$(A^+, D^{(\prime)-})$	$(A^+, C^{(\prime)-})$	$(C^+, D^{(\prime)-})$	$(A^+, A^{(\prime)-})$	$(C^+, C^{(\prime)-})$	$(D^+, D^{(\prime)-})$	$(C^+, A^{(\prime)-})$	$(D^+, C^{(\prime)-})$	$(D^+, A^{(\prime)-})$
Level 0	9.92(36)	0.0	0.0	0.0	1.0	0.0	0.0	0.0	0.0	0.0
Level 1	3.74(50)	0.0	0.05	0.0	0.94	0.0	0.0	0.0	0.0	0.0
Level 2	1.95(61)	0.0	0.13	0.02	0.66	0.03	0.0	0.0	0.0	0.0

Table S6: **Details of the inferred structures for the TwitterSG by nested WSBM.** The table shows hierarchical community structure results for consensus partition and posterior sampling (Methods 4.3). The consensus columns report the effective number of groups B_e, and the frequency of interaction types from the consensus partition. The sampling columns report the mean and standard deviation of the effective number of groups $\langle B_e \rangle$ and standard deviation, and the certainty of the interaction types. We note that the posterior distribution of partitions exhibits a diffuse structure, suggesting multiple competing explanations for the network. To accurately capture this complexity, we identify and summarize the distinct modes of the distribution. Each mode represents a statistically significant, internally consistent clustering of the network's organization. The top levels of Modes 0 and 2 support balanced dominant types, while Modes 1 and 3 support unbalanced dominant types. The results in Fig. 8a show potential meso-scale structure of Modes 1 and 3.

Consensus											
Hierarchy	Mode	B_e	$(A^+, D^{(\prime)-})$	$(A^+, C^{(\prime)-})$	$(C^+, D^{(\prime)-})$	$(A^+, A^{(\prime)-})$	$(C^+, C^{(\prime)-})$	$(D^+, D^{(\prime)-})$	$(C^+, A^{(\prime)-})$	$(D^+, C^{(\prime)-})$	$(D^+, A^{(\prime)-})$
Level 0	0	5.18	0.17	0.14	0.06	0.00	0.06	0.11	0.11	0.14	0.11
	1	5.22	0.14	0.14	0.06	0.08	0.03	0.08	0.11	0.06	0.19
	2	5.17	0.20	0.16	0.00	0.07	0.09	0.16	0.07	0.09	0.07
	3	7.17	0.12	0.04	0.05	0.28	0.04	0.01	0.05	0.03	0.15
Level 1	0	1.96	1.00	0.00	0.00	0.00	0.00	0.00	0.00	0.00	0.00
	1	2.04	0.33	0.00	0.00	0.00	0.00	0.33	0.00	0.00	0.00
	2	1.99	0.67	0.00	0.00	0.33	0.00	0.00	0.00	0.00	0.00
	3	5.38	0.10	0.10	0.00	0.29	0.14	0.00	0.19	0.00	0.05
Level 2	0	1.00	0.00	0.00	0.00	0.00	0.00	0.00	0.00	0.00	0.00
	1	1.97	0.00	0.00	0.00	0.00	1.00	0.00	0.00	0.00	0.00
	2	1.95	1.00	0.00	0.00	0.00	0.00	0.00	0.00	0.00	0.00
	3	2.63	0.00	0.00	0.00	0.33	0.67	0.00	0.00	0.00	0.00
Sampling											
Hierarchy	Mode	$\langle B_e \rangle$	$(A^+, D^{(\prime)-})$	$(A^+, C^{(\prime)-})$	$(C^+, D^{(\prime)-})$	$(A^+, A^{(\prime)-})$	$(C^+, C^{(\prime)-})$	$(D^+, D^{(\prime)-})$	$(C^+, A^{(\prime)-})$	$(D^+, C^{(\prime)-})$	$(D^+, A^{(\prime)-})$
Level 0	0	8.91(29)	0.65	0.00	0.00	0.00	0.00	0.00	0.00	0.00	0.34
	1	9.00(00)	0.27	0.02	0.00	0.00	0.00	0.27	0.06	0.00	0.38
	2	9.00(00)	0.65	0.01	0.00	0.00	0.00	0.00	0.00	0.01	0.33
	3	13.0(00)	0.00	0.00	0.00	1.00	0.00	0.00	0.00	0.00	0.00
Level 1	0	2.49(64)	0.94	0.00	0.01	0.00	0.00	0.00	0.00	0.04	0.00
	1	3.22(41)	0.77	0.21	0.02	0.00	0.00	0.00	0.00	0.00	0.00
	2	3.25(52)	0.84	0.15	0.00	0.00	0.02	0.00	0.00	0.00	0.00
	3	7.02(14)	0.00	0.00	0.00	0.75	0.00	0.00	0.25	0.00	0.00
Level 2	0	1.45(54)	0.14	0.00	0.08	0.00	0.21	0.00	0.00	0.00	0.00
	1	2.02(14)	0.12	0.00	0.01	0.00	0.87	0.00	0.00	0.00	0.00
	2	2.22(47)	0.57	0.00	0.19	0.00	0.22	0.00	0.00	0.00	0.00
	3	3.06(24)	0.00	0.00	0.00	0.00	1.00	0.00	0.00	0.00	0.00

Table S7: **Details of the inferred structures for the USA Congress by nested LSBM.** The table shows hierarchical community structure results for consensus partition and posterior sampling (Methods 4.3). The consensus columns report the frequency of interaction types from the consensus partition for each fixed number of groups B. The sampling columns report the certainty of the interaction types for each fixed number of groups B. The primary interaction type is balanced, incorporating core-periphery alongside other types, which confirms the findings in Fig. 8b.

				Consensus					
Fixed B	$(A^+, D^{(')-})$	$(A^+, C^{(')-})$	$(C^+, D^{(')-})$	$(A^+, A^{(')-})$	$(C^+, C^{(')-})$	$(D^+, D^{(')-})$	$(C^+, A^{(')-})$	$(D^+, C^{(')-})$	$(D^+, A^{(')-})$
2	0.71	0.11	0.04	0.01	0.10	0.0	0.03	0.01	0.0
3	0.68	0.14	0.02	0.01	0.10	0.10	0.04	0.0	0.0
4	0.68	0.13	0.04	0.0	0.13	0.0	0.03	0.0	0.0
5	0.71	0.13	0.02	0.0	0.11	0.0	0.03	0.0	0.0
				Sampling					
Fixed B	$(A^+, D^{(')-})$	$(A^+, C^{(')-})$	$(C^+, D^{(')-})$	$(A^+, A^{(')-})$	$(C^+, C^{(')-})$	$(D^+, D^{(')-})$	$(C^+, A^{(')-})$	$(D^+, C^{(')-})$	$(D^+, A^{(')-})$
2	0.70	0.12	0.03	0.01	0.10	0.0	0.04	0.0	0.0
3	0.70	0.13	0.03	0.01	0.10	0.0	0.04	0.0	0.0
4	0.71	0.12	0.04	0.0	0.11	0.0	0.03	0.0	0.0
5	0.70	0.13	0.03	0.01	0.10	0.0	0.03	0.0	0.0

Table S8: **Details of the inferred structures for the CoW by nested WSBM.** The table shows hierarchical community structure results for consensus partition and posterior sampling (Methods 4.3). The consensus columns report the effective number of groups B_e, and the frequency of interaction types from the consensus partition. The sampling columns report the mean and standard deviation of the effective number of groups $\langle B_e \rangle$ and standard deviation, and the certainty of the interaction types. The dominant type of Level 1 is $(A^+, C^{(')-})$, which confirms the results in Fig. 8c.

					Consensus					
Hierarchy	B_e	$(A^+, D^{(')-})$	$(A^+, C^{(')-})$	$(C^+, D^{(')-})$	$(A^+, A^{(')-})$	$(C^+, C^{(')-})$	$(D^+, D^{(')-})$	$(C^+, A^{(')-})$	$(D^+, C^{(')-})$	$(D^+, A^{(')-})$
Level 0	4.72	0.2	0.1	0.1	0.3	0.1	0.1	0.0	0.0	0.0
Level 1	2.89	0.0	0.67	0.33	0.0	0.0	0.0	0.0	0.0	0.0
					Sampling					
Hierarchy	$\langle B_e \rangle$	$(A^+, D^{(')-})$	$(A^+, C^{(')-})$	$(C^+, D^{(')-})$	$(A^+, A^{(')-})$	$(C^+, C^{(')-})$	$(D^+, D^{(')-})$	$(C^+, A^{(')-})$	$(D^+, C^{(')-})$	$(D^+, A^{(')-})$
Level 0	4.68(20)	0.19	0.03	0.0	0.78	0.0	0.0	0.0	0.0	0.0
Level 1	2.81(35)	0.0	0.76	0.01	0.22	0.0	0.01	0.0	0.0	0.0

S3 Derivation of Eq. (11)

To derive the description length of the uncorrelated edge signs, $S(W)$, we model the sign of each undirected edge as an independent, identically distributed random variable governed solely by the global probability of a positive edge, p. Given the observed network W, the conditional likelihood is

$$P(W|p) = \prod_{ij\in E} p^{\delta_{W_{ij},+1}}(1-p)^{\delta_{W_{ij},-1}} = p^{L^+}(1-p)^{L^-}, \tag{S1}$$

where L^+ is the number of positive edges, L^- is the number of negative edges, and $L = L^+ + L^-$ is the total number of undirected edges.

In the Minimum Description Length (MDL) framework, we avoid fitting a specific point estimate for p and instead compute a marginal likelihood. Assuming a uniform, non-informative prior $P(p) = 1$ for $p \in [0, 1]$, we integrate over p to obtain

$$P(W) = \int_0^1 p^{L^+}(1-p)^{L^-}\,dp. \tag{S2}$$

This integral is exactly the Beta function (α, β), with parameters $\alpha = L^+ + 1$ and $\beta = L^- + 1$. Expressing this via the Gamma function, $\Gamma(n) = (n-1)!$, yields

$$P(W) = \frac{\Gamma(L^+ + 1)\Gamma(L^- + 1)}{\Gamma(L+2)} = \frac{L^+!L^-!}{(L+1)!}. \tag{S3}$$

To simplify, we expand the denominator as $(L + 1)! = (L + 1)L!$ and recognize the inverse of the binomial coefficient, $\binom{L}{L^+}^{-1} = \frac{L^+!L^-!}{L!}$, leading to

$$P(W) = \frac{1}{(L+1)\binom{L}{L^+}}. \tag{S4}$$

Finally, Eq. (11) is recovered computing $S(W) = -\ln P(W)$.

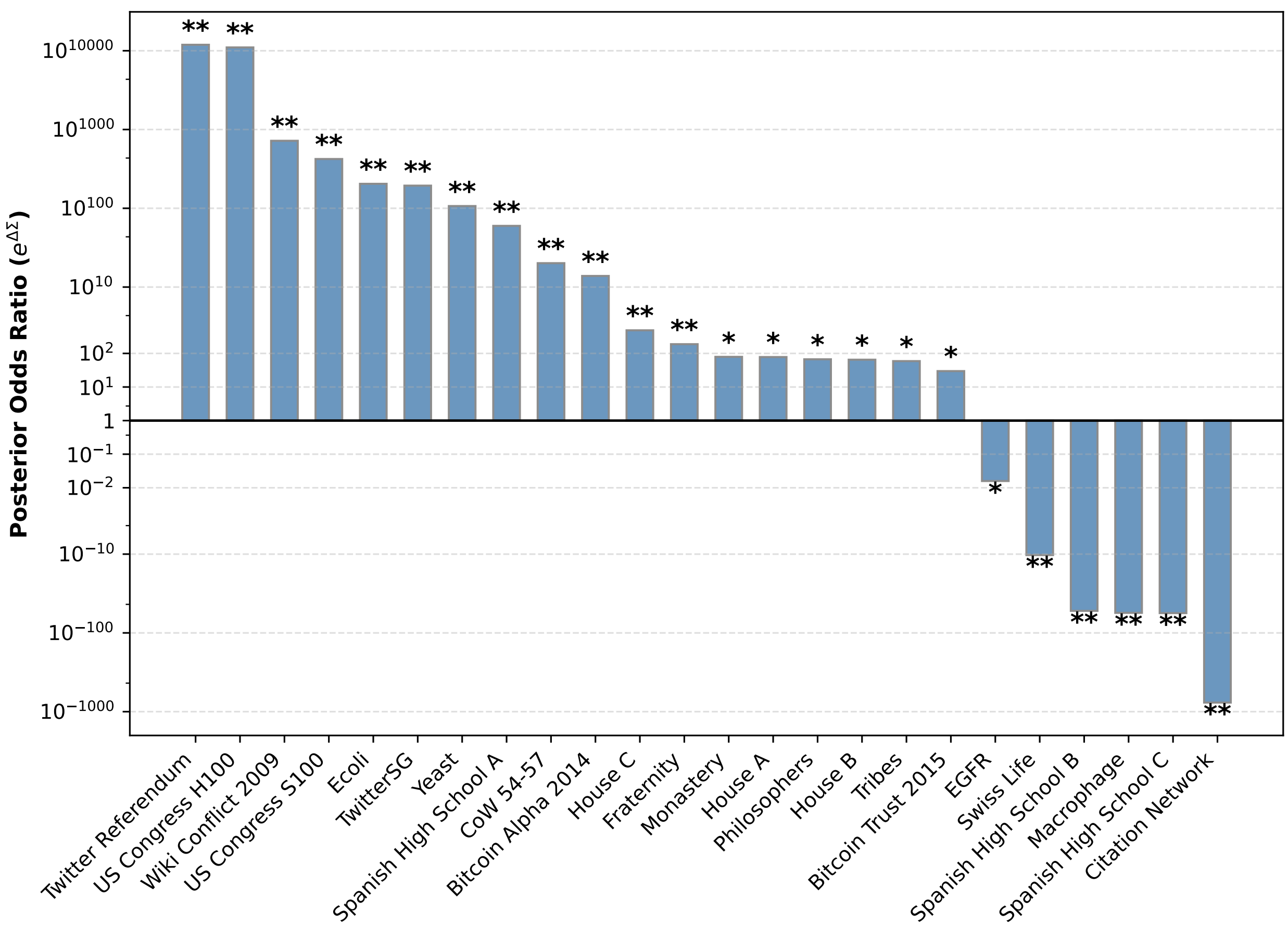

Figure S7: **Structural information gained by incorporating edge signs across empirical networks.** The y-axis displays the posterior odds ratio ($e^{\Delta\Sigma}$), quantifying how many times more likely the signed generative model is compared to the unsigned null hypothesis for the entire network (see Methods Sec. 4.6). A symmetric log-scaled y-axis is utilized to ensure visual balance between positive evidence (ratios > 1, favoring the signed model) and negative evidence (ratios < 1, favoring the unsigned hypothesis), centered around a solid baseline at 1. Across the majority of datasets, edge signs act as a highly compressible generative signal. To highlight the statistical significance, datasets are marked with a single asterisk ($*$) if the posterior odds ratio favors either model by a factor exceeding 10 ($e^{|\Delta\Sigma|} > 10$), and a double asterisk ($**$) if the factor exceeds 100 ($e^{|\Delta\Sigma|} > 100$).

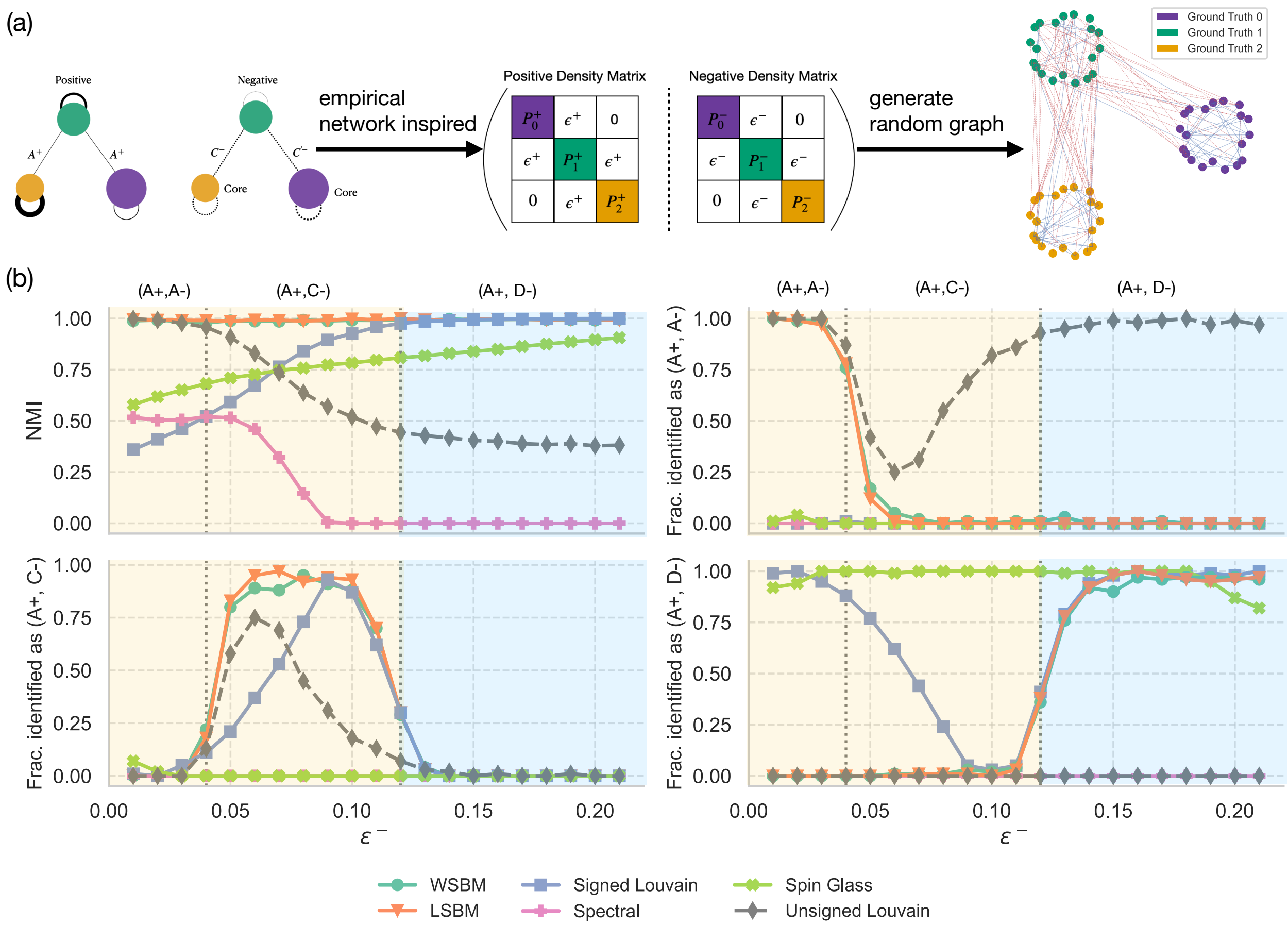

Figure S8: **Heuristic methods lead to biased partitions in unbalanced synthetic networks.** (a) We generate networks from a model consisting of three groups of equal size ($N/3 = 60$ nodes each), with block probability matrices inspired by the empirical density matrices of CoW studied in Fig. 8: intra-group positive probabilities are asymmetric across blocks $P_i^+ = (0.16, 0.08, 0.16)$, intra-group negative probabilities are non-negligible $P_i^- = (0.04, 0.12, 0.04)$, inter-group positive probability is fixed at $\epsilon^+ = 0.01$, and variable inter-group negative probability ϵ^-. For $\epsilon^- < 0.04$ the network is dominated by pure assortative $(A^+, A^{(')-})$ relationships, for $0.04 < \epsilon^- < 0.12$ by the unbalanced $(A^+, C^{(')-})$ relationship, and for $\epsilon^- > 0.12$ by the balanced relationship $(A^+, D^{(')-})$. (b) Performance of different methods as a function of ε^-. The four plots show the normalized mutual information (NMI) between detected and ground-truth partitions and fraction of networks in which each method identifies the dominant interaction type as $(A^+, A^{(')-})$, $(A^+, C^{(')-})$, and $(A^+, D^{(')-})$. Results are averaged across 100 independent random networks. The two structural transition are marked by vertical dotted lines, at $\epsilon^- = 0.04$ and $\epsilon^- = 0.12$. Across all regimes, both SBMs correctly track the ground truth, maintaining high NMI throughout. Signed Louvain performs well only in the $(A^+, D^{(')-})$ regime. (Unsigned) Louvain performs well only in the $(A^+, A^{(')-})$ regime, where ignoring edge signs does not obscure the community structure. Both Louvain and Signed Louvain do not work well in the $(A^+, C^{(')-})$ regime.

REFERENCES

1. B. Ordozgoiti, A. Matakos, A. Gionis, Finding large balanced subgraphs in signed networks. *Proceedings of The Web Conference 2020* (ACM, 2020), pp. 1378–1388.

2. M. Ruiz-Garcia, J. Ozaita, M. Pereda, A. Alfonso, P. Branas-Garza, J. A. Cuesta, A. Sanchez, Triadic influence as a proxy for compatibility in social relationships. *Proc. Natl. Acad. Sci. U.S.A.* **120**, e2215041120 (2023).

3. J. Pougué-Biyong, A. Gupta, A. Haghighi, A. El-Kishky, Learning stance embeddings from signed social graphs. *Proceedings of the Sixteenth ACM International Conference on Web Search and Data Mining* (ACM, 2023), pp. 177–185.

4. S. Talaga, M. Stella, T. J. Swanson, A. S. Teixeira, Polarization and multiscale structural balance in signed networks. *Commun. Phys.* **6**, 349 (2023).

5. B. Hao, I. A. Kovács, Proper network randomization is key to assessing social balance. *Sci. Adv.* **10**, eadj0104 (2024).

6. A. Gallo, D. Garlaschelli, T. Squartini, Assessing frustration in real-world signed networks: A statis-tical theory of balance. *Phys. Rev. Res.* **6**, L042065 (2024).

7. A. Gallo, F. Saracco, R. Lambiotte, D. Garlaschelli, T. Squartini, Patterns of link reciprocity in directed, signed networks. *Phys. Rev. E* **111**, 024312 (2025).

8. F. Diaz-Diaz, E. Candellone, M. A. González-Casado, E. Fraxanet, A. Vendeville, I. Ferri, A. S. Teixeira, Signed Networks: Theory, methods, and applications. arXiv [physics.soc-ph] (2025).

9. M. A. González-Casado, A. Sánchez, S. Fortunato, What defines a group of friends? Rethinking community structure in signed, directed networks. arXiv [physics.soc-ph] (2025).

10. S. Fortunato, Community detection in graphs. *Phys. Rep.* **486**, 75–174 (2010).

11. V. A. Traag, J. Bruggeman, Community detection in networks with positive and negative links. *Phys. Rev. E* **80**, 036115 (2009).

12. P. J. Mucha, T. Richardson, K. Macon, M. A. Porter, J.-P. Onnela, Community structure in time-dependent, multiscale, and multiplex networks. *Science* **328**, 876–878 (2010).

13. T. P. Peixoto, Inferring the mesoscale structure of layered, edge-valued, and time-varying networks. *Phys. Rev. E* **92**, 042807 (2015).

14. D. Cartwright, F. Harary, Structural balance: A generalization of Heider's theory. *Psychol. Rev.* **63**, 277–293 (1956).

15. F. Heider, Attitudes and cognitive organization. *J. Psychol.* **21**, 107–112 (1946).

16. F. Harary, Structural duality. *Behav. Sci.* **2**, 255–265 (1957).

17. Y. Tian, R. Lambiotte, Spreading and structural balance on signed networks. *SIAM J. Appl. Math.* **23**, 50–80 (2024).

18. F. Harary, On the notion of balance of a signed graph. *Mich. Math. J.* **2**, 143–146 (1953).

19. P. Doreian, A. Mrvar, A partitioning approach to structural balance. *Soc. Netw.* **18**, 149–168 (1996).

20. G. Facchetti, G. Iacono, C. Altafini, Computing global structural balance in large-scale signed social networks. *Proc. Natl. Acad. Sci. U.S.A.* **108**, 20953–20958 (2011).

21. S. Aref, L. Dinh, R. Rezapour, J. Diesner, Multilevel structural evaluation of signed directed social networks based on balance theory. *Sci. Rep.* **10**, 15228 (2020).

22. R. Singh, B. Adhikari, Measuring the balance of signed networks and its application to sign prediction. *J. Stat. Mech. Theory Exp.* **2017**, 063302 (2017).

23. S. Aref, M. C. Wilson, Balance and frustration in signed networks. *J. Complex Netw.* **7**, 163–189 (2019).

24. J. Kunegis, S. Schmidt, A. Lommatzsch, J. Lerner, E. W. De Luca, S. Albayrak, Spectral analysis of signed graphs for clustering, prediction and visualization. *Proceedings of the 2010 SIAM International Conference on Data Mining* (SIAM, 2010), pp. 559–570.

25. M. Cucuringu, P. Davies, A. Glielmo, H. Tyagi, SPONGE: A generalized eigenproblem for clustering signed networks. *Proceedings of the 22nd International Conference on Artificial Intelligence and Statistics* (PMLR, 2019), vol. 89, pp. 1088–1098.

26. E. Estrada, M. Benzi, Walk-based measure of balance in signed networks: Detecting lack of balance in social networks. *Phys. Rev. E* **90**, 042802 (2014).

27. A. Kirkley, G. T. Cantwell, M. E. Newman, Balance in signed networks. *Phys. Rev. E* **99**, 012320 (2019).

28. M. E. Newman, M. Girvan, Finding and evaluating community structure in networks. *Phys. Rev. E* **69**, 026113 (2004).

29. A. Amelio, C. Pizzuti, Community mining in signed networks: A multiobjective approach. *Proceedings of the 2013 IEEE/ACM International Conference on Advances in Social Networks Analysis and Mining* (ACM, 2013), pp. 95–99.

30. V. A. Traag, R. Aldecoa, J.-C. Delvenne, Detecting communities using asymptotical surprise. *Phys. Rev. E* **92**, 022816 (2015).

31. J. N. Pougué-Biyong, R. Lambiotte, SignedLouvain: Louvain for signed networks. arXiv [cs.SI] (2024).

32. K.-Y. Chiang, J. J. Whang, I. S. Dhillon, Scalable clustering of signed networks using balance normalized cut. *Proceedings of the 21st ACM International Conference on Information and Knowledge Management* (ACM, 2012), pp. 615–624.

33. J. Kunegis, Applications of structural balance in signed social networks. arXiv [cs.SI] (2014).

34. J. Reichardt, S. Bornholdt, Statistical mechanics of community detection. *Phys. Rev. E* **74**, 016110 (2006).

35. G. Facchetti, G. Iacono, C. Altafini, Exploring the low-energy landscape of large-scale signed social networks. *Phys. Rev. E* **86**, 036116 (2012).

36. R. F. Betzel, M. A. Bertolero, D. S. Bassett, Non-assortative community structure in resting and task-evoked functional brain networks. bioRxiv [Preprint] 355016, (2018).

37. C. X. Liu, T. J. Alexander, E. G. Altmann, Nonassortative relationships between groups of nodes are typical in complex networks. *PNAS Nexus* **2**, pgad364 (2023).

38. T. P. Peixoto, *Descriptive vs. Inferential Community Detection in Networks* (Cambridge Univ. Press, 2023).

39. J. Q. Jiang, Stochastic block model and exploratory analysis in signed networks. *Phys. Rev. E* **91**, 062805 (2015).

40. L. Peel, D. B. Larremore, A. Clauset, The ground truth about metadata and community detection in networks. *Sci. Adv.* **3**, e1602548 (2017).

41. R. Guimerà, One model to rule them all in network science? *Proc. Natl. Acad. Sci. U.S.A.* **117**, 25195–25197 (2020).

42. L. Peel, T. P. Peixoto, M. De Domenico, Statistical inference links data and theory in network science. *Nat. Commun.* **13**, 6794 (2022).

43. T. P. Peixoto, A. Kirkley, Implicit models, latent compression, intrinsic biases, and cheap lunches in community detection. *Phys. Rev. E* **108**, 024309 (2023).

44. B. Karrer, M. E. Newman, Stochastic blockmodels and community structure in networks. *Phys. Rev. E* **83**, 016107 (2011).

45. T. P. Peixoto, Nonparametric weighted stochastic block models. *Phys. Rev. E* **97**, 012306 (2018).

46. T. P. Peixoto, “Bayesian stochastic blockmodeling” in *Advances in Network Clustering and Blockmodeling*, P. Doreian, V. Batagelj, A. Ferligoj, Eds. (John Wiley & Sons Ltd., 2019), chap. 11, pp. 289–332.

47. P. Rombach, M. A. Porter, J. H. Fowler, P. J. Mucha, Core-periphery structure in networks (revis-ited). *SIAM Rev.* **59**, 619–646 (2017).

48. T. P. Peixoto, Hierarchical block structures and high-resolution model selection in large networks. *Phys. Rev. X* **4**, 011047 (2014).

49. T. P. Peixoto, The graph-tool Python library Software. 2014. https://graph-tool.skewed.de.

50. T. P. Peixoto, Merge-split Markov chain Monte Carlo for community detection. *Phys. Rev. E* **102**, 012305 (2020).

51. M. T. Rivera, S. B. Soderstrom, B. Uzzi, Dynamics of dyads in social networks: Assortative, relational, and proximity mechanisms. *Annu. Rev. Sociol.* **36**, 91–115 (2010).

52. P. Csermely, A. London, L.-Y. Wu, B. Uzzi, Structure and dynamics of core/periphery networks. *J. Complex Netw.* **1**, 93–123 (2013).

53. M. Bastos, C. Piccardi, M. Levy, N. McRoberts, M. Lubell, Core-periphery or decentralized? Topo-logical shifts of specialized information on Twitter. *Soc. Netw.* **52**, 282–293 (2018).

54. Z. Neal, The backbone of bipartite projections: Inferring relationships from co-authorship, co-sponsorship, co-attendance and other co-behaviors. *Soc. Netw.* **39**, 84–97 (2014).

55. P. Doreian, A. Mrvar, Structural balance and signed international relations. *J. Soc. Struct.* **16**, 1–49 (2015).

56. O. Askarisichani, J. N. Lane, F. Bullo, N. E. Friedkin, A. K. Singh, B. Uzzi, Structural balance emerges and explains performance in risky decision-making. *Nat. Commun.* **10**, 2648 (2019).

57. F. Radicchi, F. N. Silva, A. Flammini, S. Fortunato, S. Kojaku, Detectability threshold in weighted modular networks. *Phys. Rev. E* **113**, 014318 (2026).

58. B. Hao, E. Platnick, I. A. Kovács, Social balance in directed networks. *Commun. Phys.* **8**, 215 (2025).

59. A. Gallo, D. Garlaschelli, R. Lambiotte, F. Saracco, T. Squartini, Testing structural balance theories in heterogeneous signed networks. *Commun. Phys.* **7**, 154 (2024).

60. T. P. Peixoto, Nonparametric Bayesian inference of the microcanonical stochastic block model. *Phys. Rev. E* **95**, 012317 (2017).

61. W. Zhang, MesoScaleSN: Code for meso-scale structures in signed networks. *Zenodo* (2026); 10.5281/zenodo.19412922.

62. T. P. Peixoto, Revealing consensus and dissensus between network partitions. *Phys. Rev. X* **11**, 021003 (2021).

63. A. L. N. Fred, A. K. Jain, Robust data clustering. *Proceedings of the 2003 IEEE Computer Society Conference on Computer Vision and Pattern Recognition* (IEEE, 2003), vol. 2, pp. II-128–II-133.

64. H. Felippe, F. Battiston, A. Kirkley, Network mutual information measures for graph similarity. *Commun. Phys.* **7**, 335 (2024).

65. M. Szell, R. Lambiotte, S. Thurner, Multirelational organization of large-scale social networks in an online world. *Proc. Natl. Acad. Sci. U.S.A.* **107**, 13636–13641 (2010).

66. K. E. Read, Cultures of the central highlands, New Guinea. Southwest. *J. Anthropol.* **10**, 1–43 (1954).

67. T. M. Newcomb, *The Acquaintance Process* (Holt, Rinehart & Winston, 1961), pp. 259–261.

68. S. F. Sampson, "A novitiate in a period of change: An experimental and case study of social relationships," thesis, Cornell University, Ithaca, NY (1968).

69. T. B. Lemann, R. L. Solomon, Group characteristics as revealed in sociometric patterns and person-ality ratings. *Sociometry* **15**, 7–90 (1952).

70. A. Vörös, Z. Boda, T. Elmer, M. Hoffman, K. Mepham, I. J. Raabe, C. Stadtfeld, The Swiss StudentLife Study: Investigating the emergence of an undergraduate community through dynamic, multidimensional social network data. *Soc. Netw.* **65**, 71–84 (2021).

71. R. Collins, *The Sociology of Philosophies: A Global Theory of Intellectual Change* (Harvard Univ. Press, 1998).

72. U. Brandes, P. Kenis, J. Lerner, D. van Raaij, Network analysis of collaboration structure in Wikipedia. *Proceedings of the 18th International Conference on World Wide Web* (ACM, 2009), pp. 731–740.

73. S. Kumar, Structure and dynamics of signed citation networks. *Proceedings of the 25th International Conference Companion on World Wide Web* (ACM, 2016), pp. 63–64.

74. D. Song, W. Wang, Y. Fan, Y. Xing, A. Zeng, Quantifying the structural and temporal character-istics of negative links in signed citation networks. *Inf. Process. Manag.* **59**, 102996 (2022).

75. G. Iacono, F. Ramezani, N. Soranzo, C. Altafini, Determining the distance to monotonicity of a biological network: A graph-theoretical approach. *IET Syst. Biol.* **4**, 223–235 (2010).

76. S. Kumar, F. Spezzano, V. S. Subrahmanian, C. Faloutsos, Edge weight prediction in weighted signed networks. *2016 IEEE 16th International Conference on Data Mining (ICDM)* (IEEE, 2016), pp. 221–230.
